# The Role of Host and Microbial Factors in the Pathogenesis of Pneumococcal Bacteraemia Arising from a Single Bacterial Cell Bottleneck

**DOI:** 10.1371/journal.ppat.1004026

**Published:** 2014-03-20

**Authors:** Alice Gerlini, Leonarda Colomba, Leonardo Furi, Tiziana Braccini, Ana Sousa Manso, Andrea Pammolli, Bo Wang, Antonio Vivi, Maria Tassini, Nico van Rooijen, Gianni Pozzi, Susanna Ricci, Peter W. Andrew, Uwe Koedel, E. Richard Moxon, Marco R. Oggioni

**Affiliations:** 1 LAMMB, Department of Biotechnology, University of Siena, Siena, Italy; 2 Department of Pathophysiology, Experimental Medicine and Public Health, University of Siena, Siena, Italy; 3 Department of Mathematics, University of Leicester, Leicester, United Kingdom; 4 Centro NMR, University of Siena, Siena, Italy; 5 Department of Molecular Cell Biology, Vrije Universiteit Medical Center, Amsterdam, The Netherlands; 6 UOC Batteriologia, Azienda Ospedaliera Universitaria Senese, Siena, Italy; 7 Department of Infection, Immunity and Inflammation, University of Leicester, Leicester, United Kingdom; 8 Department of Neurology, Ludwig-Maximilians University of Munich, München, Germany; 9 Division of Medical Sciences, John Radcliffe Hospital, University of Oxford, Oxford, United Kingdom; 10 Department of Genetics, University of Leicester, Leicester, United Kingdom; Emory University, United States of America

## Abstract

The pathogenesis of bacteraemia after challenge with one million pneumococci of three isogenic variants was investigated. Sequential analyses of blood samples indicated that most episodes of bacteraemia were monoclonal events providing compelling evidence for a single bacterial cell bottleneck at the origin of invasive disease. With respect to host determinants, results identified novel properties of splenic macrophages and a role for neutrophils in early clearance of pneumococci. Concerning microbial factors, whole genome sequencing provided genetic evidence for the clonal origin of the bacteraemia and identified SNPs in distinct sub-units of F0/F1 ATPase in the majority of the *ex vivo* isolates. When compared to parental organisms of the inoculum, ex-vivo pneumococci with mutant alleles of the F0/F1 ATPase had acquired the capacity to grow at low pH at the cost of the capacity to grow at high pH. Although founded by a single cell, the genotypes of pneumococci in septicaemic mice indicate strong selective pressure for fitness, emphasising the within-host complexity of the pathogenesis of invasive disease.

## Author Summary

Decades of research on bacterial sepsis have been devoted to analysing the steps that lead from a local event, either carriage or a localised infection, to systemic disease. Our work analyses in depth the events determining systemic infection by one of the main human pathogens, *Streptococcus pneumoniae*. Consistent with similar findings on the pathogenesis of bacteraemia due to other commensal pathogens, our results show that after an intravenous inoculum of a million pneumococci, the resulting septicaemia is often founded by a single bacterial cell. Investigation into the nature of this monoclonal infection identified strong within-host selective pressure for metabolic fitness during outgrowth of the bacterial population.

## Introduction

*Streptococcus pneumoniae*, one of the major human bacterial pathogens, is also part of the normal upper respiratory tract flora, where nasopharyngeal colonisation with one or more strains often lasts weeks to months with seasonal peaks in late winter [1], [2]. Carriage of *S. pneumoniae* (pneumococci) may result in disease as the consequence of contiguous spread from the nasopharynx to other sites in the upper or lower respiratory tract causing, for example, otitis media or pneumonia. More rarely, there is hematogenous dissemination of pneumococci resulting in septicaemia and metastatic disease such as meningitis [1], [3]–[5]. In experimental models of pneumococcal infection, the challenge dose required to induce disease is dependent on the route of infection, the genetic background of the host and the virulence of the infecting strain [6] and may vary from a very few to millions of organisms [7]. Following intravenous inoculation of mice with laboratory grown pneumococci, a hallmark of experimental bacteraemic infections is the rapid and efficient clearance of most of the inoculated bacteria [8]–[10]. In non-immune rodents, major factors mediating this clearance are splenic macrophages and complement mediated opsonisation [11]–[14]. A challenge dose of about one million virulent, encapsulated pneumococci is generally needed to induce bacteraemia in about half of challenged animals (the effective dose or ED_50_) and which is the dose generally used to address investigations into the early events shaping an infectious process.

Most work on the pathogenesis of infectious disease focuses on specific virulence determinants which are generally presented as the cause, either alone or in combination with other factors, of the events leading to the infection of the host where the microbial population is considered to be a uniform entity. However, several investigations have addressed the within host population dynamics, especially on the early phases of host-pathogen interactions [15]. There are different models which address these early events that include: (i) the model of independent action, which postulates that at the LD_50_ (lethal dose for 50% for the hosts) the hosts develop infection “following the multiplication of only one of the inoculated bacteria, regardless of the total number of bacteria inoculated” [16], (ii) the hypothesis of synergy which “postulates that inoculated bacteria co-operate and that fatal infections will be initiated by more than one bacterium and that this will lead to the predominance of several variants” [16], and models which introduce time as a factor into the process and propose a two-stage model where a birth–death phase would be responsible for generation of the heterogeneity within the population later during the infection [17].

Both for viral and bacterial infections it has been shown that the effective number of infectious agents which actually start the disease is generally many orders of magnitude below the actual dose used for challenge [16], [16], [18]–[21]. In particular a series of reports, generally based on experimental challenge using an inoculum containing an approximately equal mixture of two isogenic variants at the LD50, has shown experimentally that systemic infections may be initiated by the multiplication of as few as a single organism [22]–[27]. Models differ widely, and due to the nature of pathogenesis of many infections, they rely on experimental challenge at a site different to that investigated for disease implying that multiple bottlenecks occur and that potentially a series of invasive events could be enucleated [16], [18]–[27]. We here have investigated the host and microbial determinants that underpin the occurrence of the single cell bottleneck in the pathogenesis of pneumococcal septicaemia following inoculation of mice with three isogenic variants by the intravenous route. This route has an advantage over other experimental infection models as there are less biological events between the initial challenge and full blown disease so that rigorous analysis of the events is facilitated.

## Results

### The population of pneumococci in the blood is clonal within hours of inoculation

**Figure 1:**
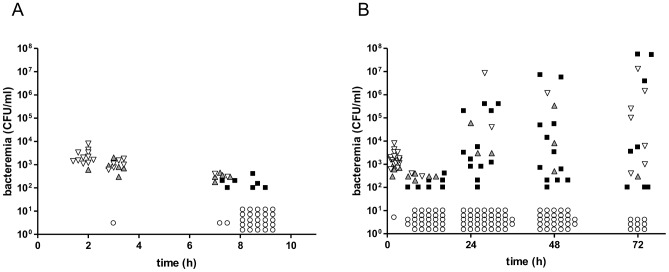
Co-infection of CD1 mice with three isogenic variants of *S. pneumoniae* TIGR4. A mixture of three isogenic *S. pneumoniae* TIGR4 variants (3×10^5^ CFU/each strain) was given to CD1 mice (n = 68) by the i.v. route. Bacterial counts were performed collecting blood at various time points. (A) Blood counts in the first 10 h after challenge. (B) Blood counts up to 72 h post-challenge, (including data reported in A). Each symbol indicates a single mouse. Blood cultures yielding all three variants are shown in downward white triangles, those yielding two variants as upward grey triangles, and samples yielding a monoclonal blood culture are shown as black squares. Samples from mice with negative blood cultures are shown as open circles. The ratio of infected over un-infected mice was 0.31 at 24 h, 0.37 at 48 h and 0.67 at 72 h. The cut off for detection is 100 CFU/ml. Data of two independent experiments are reported.

CD1 mice were inoculated intravenously (i.v.) with a mixture of pneumococci comprising approximately equal numbers of each of three isogenic TIGR4 mutants (FP122, FP321 and FP318) with different resistance markers (Table 1) [28]. Following inoculation, blood samples were collected at different times and spread on selective plates. Colony counts allowed quantitation of the distribution of the different mutants making up the pneumococcal population in the blood (Figure 1). Two hours after bacterial challenge, blood samples from all mice, with one exception, grew all three of the variants that had been included in the challenge dose. Samples of the second group of mice, sampled one hour thereafter, showed a mixed population of the variants: two in 6 mice, three in 17 mice and one mouse had negative blood culture (Figure 1A). At 7 h after challenge, this pattern was distinctly different: there were 10 positive and 2 negative blood cultures and the numbers of bacteria were significantly reduced. At 8–9 h post-infection, most blood cultures (25/29 mice) were negative. The remaining 4 mice had monoclonal blood cultures in that each grew colonies of only one mutant (Figure 1A). In the subsequent hours of infection, bacteria were detected in the blood at high concentrations (up to 1×10^6^ CFU) in 17/55 (31%) mice at 24 h, 16/43 (37%) mice at 48 h and 15/24 (62%) mice at 72 h (Figure 1B). At all these time points, most blood samples yielded colonies of only one of the variants: 12 out of 17 blood cultures at 24 h, 12/16 at 48 h and 8/16 at 72 h. Among the 32 single-variant blood samples each variant was more or less equally represented as the progenitor, although owing to the smaller challenge dose of strain FP318 in one experiment, this strain was recovered at lower density from the blood and caused fewer single-variant infections (Table S1). In three of twelve mice for which three serial blood cultures were taken we observed an increase in the number of variants. One of these mice showed evidence of infection with 3 variants at 48 h after earlier having a monoclonal infection and a further two mice had three variants infection at 72 h after having previously had infection with only one or two variants respectively. Seven of the single-variant bacteraemia isolates were checked for colony morphology and each was found to have the opaque phenotype, in contrast to the challenge strains (not-mouse-passaged) that yielded a mixed population of about similar proportions of opaque and translucent colonies [29].

**Table 1:** Pneumococcal strains.

Strain	Background	Type	Comment	Reference
D39	wt	2		[62]
DP1004	D39	rough	Un-encapsulated, streptomycin resistant	[51]
G54	wt	19F	erythromycin and tetracycline resistant	[50]
TIGR4	wt	4		[63]
FP122	TIGR4	4	*zmpC::ermB*	[28], [48]
FP318	TIGR4	4	*zmpC::aad9*	this work
FP321	TIGR4	4	*zmpC::aphIII*	this work
FP335	D39	2	*bglA::aad9*	gift of Hasan Yesilkaya
FP487	FP318	4	SP1507 *atpC* mutant*	this work
FP489	FP321	4	SP1508 *atpD* mutant*	this work
FP490	FP321	4	SP1513 *atpB* mutant*	this work
FP498	FP321	4	SP1508 *atpD* mutant*	this work
FP499	DP1004	Rough	SP1507 *atpC* mutant*	this work
FP500	DP1004	Rough	SP1507 *atpC* mutant*	this work
FP503	FP321	4	SP1510 *atpA* mutant*	this work
FP504	FP321	4	SP1510 *atpA* mutant*	this work
FP505	FP318	4	SP1508 *atpD* mutant*	this work
FP506	FP321	4	SP1509 *atpG* mutant*	this work

*see Table 3 for detailed description.

The bacterial counts from cultures of spleen tissue were assayed in twelve mice at each time point (Figure S1). All mice which had positive blood cultures showed bacterial counts also in splenic samples. In addition, small numbers of organisms were cultured from spleen tissue both at 24 and 48 h in two mice each which presented with sterile blood cultures. Similarly at 72 h, bacteria were only detected in the spleen in one mouse (Figure S1). These data show that infectious foci can be detected in the spleens of mice that have negative blood cultures, indicating that the spleen is the probable site where the infection originates.

### Statistical analysis shows that bacteraemia is independently founded by a single organism

Our data indicate that the near totality of bacteria in the challenge with three isogenic pneumococcal variants is cleared by the immune system (predominantly by splenic macrophages; see below) and that few bacterial cells remain viable within a defined site of the host (i.e. the spleen). This small number of bacteria may start to grow and re-invade the host giving rise to bacteraemia. In our experimental infection most of the mice challenged were not bacteraemic and, of those becoming bacteraemic, most were infected by a single bacterial variant. In addition, we detected in some mice an increase in variants within blood cultures over time. These data indicate that over time more than one invasion event may occur. Theoretically bacteraemia may be generated in two ways: (i) by a single bacterium establishing a population in the blood in a single invasion event or several bacteria each independently establishing a population in distinct invasion events (independent action); (ii) by a defined number (more than one) of bacteria acting together to invade once or several times (co-operative synergism) [22]. We hypothesise that the former explanation pretains.

To statistically evaluate the number of bacteria involved in founding the blood population in each invasion event, we construct a model that assumes that bacteria invade and establish a population in the blood at random (Supplementary text) [23]. In this model, the number of invasion events in each mouse is assumed to follow a Poisson distribution so the expected number can be estimated from the proportion of the non-bacteraemic mice. Then we determined the expected numbers of mice infected with one, two or three variants, assuming that the number of bacteria (w) responsible for establishing the blood population in each invasion event were 1, 2, 3, etc (Table 2). Given that some mice were culled during the course of the experiment and some got multiple samplings, we limited the statistical analysis to the observations at the 24 h time point. In table 2, we report the comparison between the expected versus the observed numbers of the infected mice with one, two and three variants for different numbers of bacteria (w) potentially responsible for founding a population blood. The statistical analysis shows that the most probable number of bacteria responsible for establishing a blood population is 1 (Table 2). Note that the p-value was calculated by combining data for blood cultures with two or three variants because of the small expected frequencies in these two categories. Given that w is equal to one, we conclude that polyclonal blood infections are the result of more than one invasion event, each event founded by a single bacterium, consistent with the observed time-dependent increase in the frequency of polyclonal bacteremia over the 72 h of the experiment.

**Table 2:** Statistical analysis: number of blood cultures with one, two or three pneumococcal variants observed at 24 h in the experimental infection compared to those predicted from the statistical analysis.

		total	one	two	three	
**Observed infection outcome**		17	12	3	2	
**Predicted infection outcome**						***P***
	**w = 1**	17	14.91	1.99	0.07	**0.10**
	**w = 2**	17	4.76	10.78	1.42	0.00
	**w = 3**	17	1.57	10.03	5.37	0.00
	**w = 4**	17	0.52	7.57	8.88	0.00

### Depletion of macrophages and neutrophils defines the key role of innate cellular immunity in early clearance of bacteraemia

**Figure 2:**
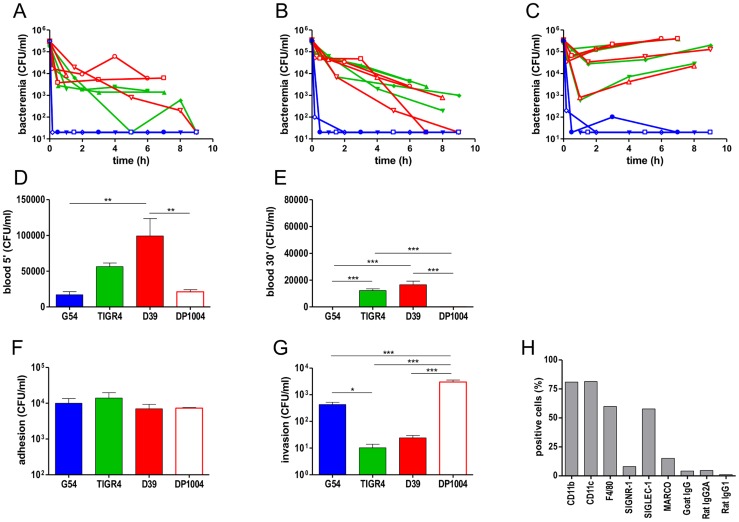
Pneumococcal sepsis and phagocytosis by splenic macrophages. Intravenous challenge of four outbred CD1 mice (A),four inbred BALB/c mice (B) and four inbred CBA/Ca mice (C) was performed by co-infection with D39 (red), G54 (blue) and TIGR4 (green). Bacterial counts in blood collected at different time points from each single mouse are reported (A–C). Cut off is 100 CFU/ml). Blood counts at 5 min (D) and 30 min (E) after i.v. infection of BALB/c mice (n = 6–12) with four pneumococcal strains: D39 (red bar), G54 (blue bar), TIGR4 (green bar) and a rough D39 derivative DP1004 (red open bar). Adhesion (F) and invasion (phagocytosis) (G) of pneumococcal strains in primary spleen macrophages from BALB/c mice. Independent experiments were run in triplicate and reported as mean ± SEM. Statistical analysis were performed with Kruskal-Wallis and Dunn's test. (H) Cytoflurimetric characterisation of surface markers of the primary spleen macrophages. A representative experiment is reported. Comparable data on splenic macrophages isolates from C57BL/6 mice are in Figure S4.

Prior to the assessment of the impact of host factors in the control of bacteraemia, we compared bacterial counts in the blood of two mouse strains known to be resistant to pneumococcal infection (outbred CD1 mice and inbred BALB/c mice) and a susceptible mouse strain CBA/Ca [30]. The mice were inoculated i.v. with a mixture of three encapsulated pneumococcal strains of different serotypes, D39 (type 2), TIGR4 (type 4) and G54 (type 19F). Bacterial clearance in CD1 and BALB/c mice showed similar kinetics (Figure 2 A–B), while CBA/Ca mice were less able to reduce the initial number of bacteria (Figure 2 C). All mouse strains cleared G54 bacteria immediately (no positive blood culture 10 min after infection) and showed a first phase of rapid clearance also for both D39 and TIGR4. Only the two resistant mouse strains showed bacterial numbers in the blood that were less than the limit of detection. In contrast, bacterial numbers increased in the susceptible strain after the first phase (Figure 2 A–C). This indicates that, depending on which host-pathogen pair was investigated, the bottlenecks may vary considerably. Since BALB/c mice showed a more uniform clearance of bacteria, subsequent experiments were conducted with this mouse strain in order to keep experimental groups to a minimal size.

To identify the host immune cells responsible for the initial clearance of bacteria from the blood, we performed a set of experiments in BALB/c mice depleted either of macrophages or neutrophils. Macrophage depletion was achieved by intraperitoneal (i.p.) injection of clodronate liposomes and neutrophil depletion by using anti-GR-1 monoclonal antibody [31]–[33]. Control groups were treated either with PBS-containing liposomes as control for clodronate experiments or with isotype-matched antibody in the case of experiments with anti-GR-1. The results obtained for the control groups were comparable to the untreated control mice and differed from the groups of mice treated with clodronate (Figure S3 A–C) or anti-GR-1 (Figure S3 E–G). To verify macrophage or neutrophils depletion, spleen samples were analyzed by flow cytometry. The reduction of macrophages in the spleen of clodronate-treated mice was 61%±14.2 measure by anti-F4/80 and 47%±10.4 by anti-CD11b compared to naïve mice. Similar results were obtained when liver samples were analyzed (Figure S3 D). In anti-GR-1-treated mice, the neutrophil number was reduced by 83%±2.7 as compared to control mice (Figure S3 H). To check for anti-pneumococcal antibodies in naïve mice, we evaluated the reactivity of mouse serum towards whole pneumococcal cells. Mice had no detectable serum antibodies to any of the pneumococcal serotypes (Figure S3 I–K). A result supported by the observation that addition of type specific rabbit serum to the blood from naïve mice conferred specific bactericidal activity (*P*<0.01).

**Figure 3:**
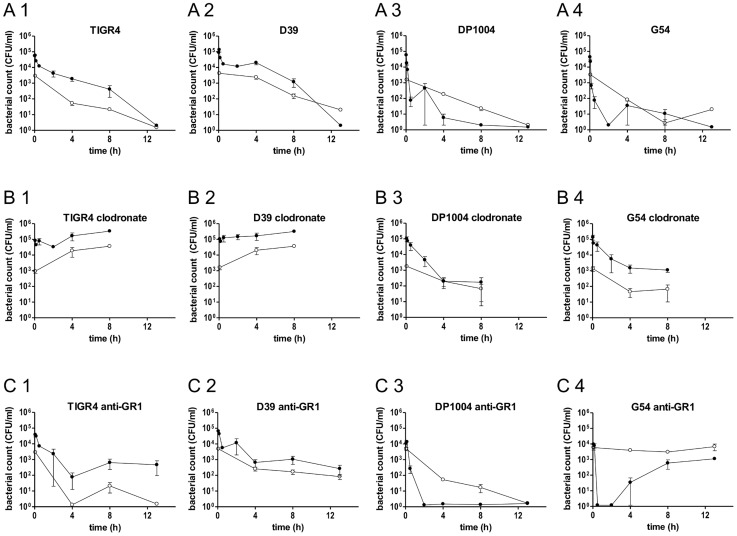
Bacterial counts in blood and spleen of BALB/c mice depleted of neutrophils or macrophages. Twelve groups of BALB/c mice (n = 3–9) were infected by the i.v. route with four different strains of *S. pneumoniae* (TIGR4, D39, DP1004 and G54) at the challenge dose of 2.5×10^5^ CFU/each strain. Bacterial counts over time of each pneumococcal strain in the blood (black lines) and in the spleen (grey lines) were reported for untreated mice (A1, A2, A3 and A4), clodronate liposomes treated mice (B1, B2, B3 and B4) and anti-GR1 mAb treated mice (C1, C2, C3 and C4). Samples were collected over 13 h, with the exception of mice treated with clodronate (8 h). The cut off is 20 CFU/ml. Data are reported as the mean ± SD of bacterial counts.

To analyse the role of macrophages and neutrophils, mice were divided into three groups: untreated (Figure 3 A1–A4), clodronate-treated (Figure 3 B1–B4), and anti-GR1-treated (Figure 3 C1–C4). After i.v. challenge with a mixture of four different strains (TIGR4, D39, DP1004 and G54), time course of bacterial counts was monitored by sampling blood, spleen, lung, liver and kidney (Figure 3 and S2). Analysis of control mice allowed categorisation of the pneumococcal strains into two groups: TIGR4 and D39, which were slowly cleared (virulent strains), and G54 and DP1004, which were cleared from the blood within minutes. The counts of TIGR4 and D39 were higher in the blood than in the spleen at 5 min and at 4 h compared to the other two strains (*P*<0.05). Bacterial loads in the other organs were similar to those found in the spleen (Figure S2). In contrast, mice infected with strains DP1004 and G54 showed higher CFU counts in the spleen than in the blood and other organs (*P*<0.05 at 5 min for both strains, *P*<0.01 at 4 and 8 h for DP1004) (Figure 3 A3–4).

The groups of mice depleted of macrophages showed significantly reduced ability to clear bacteria from the bloodstream. An increase in bacterial numbers in blood from 5 min to the later time points was observed in mice infected with TIGR4 (Figure 3 B1) and D39 (Figure 3 B2) (*P*<0.01). Blood bacterial counts were significantly higher in the clodronate-treated mice than in the control group (*P*<0.05 for all time points for both D39 and TIGR4). Bacterial counts of TIGR4 and D39 in liver and spleen were lower but with a similar trend, over time, to those in the blood (Figure 3 B1–B2 and Figure S2 B1–B2). In clodronate-treated mice, the numbers of non-virulent bacteria (strains G54 and DP1004) were higher in the blood than in the spleen (*P*<0.05 at 5 min for DP1004 and *P*<0.05 at 5 and 4 h for G54) and paralleled the trend observed for the virulent strains TIGR4 and D39 in untreated animals (Figure 3 B3–B4 compared to A1–A2).

In neutrophil-depleted mice, bacterial counts of both TIGR4 (Figure 3 C1) and D39 (Figure 3 C2) in blood and spleen decreased in the first 4 h after challenge with a similar trend to that observed in untreated animals. Thereafter, blood and organ counts remained stable (Figure 3 C1–C2 and Figure S2 C1–C2). For both virulent strains, the number of bacteria were higher in the blood than in the spleen (*P*<0.01 at 5 min). Interestingly, strain G54 had a peculiar behaviour in neutrophil-depleted mice, as it persisted in the spleen at high levels throughout the whole experiment despite being cleared from the blood within a few min of infection (*P*<0.001 at 4 and 8 h), (Figure 3 C4). At 4 h post-infection G54 bacteria reappeared in the blood (Figure 3 C4). The experiment was repeated for the later time points, and the pattern of counts was identical. The rough DP1004 strain was cleared from each body site as well as in untreated mice (Figure 3 C3).

To determine more precisely the early events occurring in the clearance of pneumococci, we have plotted separately the data on blood bacterial counts obtained 5 min and 30 min after challenge (Figure 2 D–E). In untreated mice, bacterial blood counts of the invasive strains D39 and TIGR4 were respectively 6.2×10^4^ and 5.4×10^4^ CFU/ml, while those of the non-invasive strains, DP1004 and G54, were three times lower (reduction of 60 to 75%). Differences between the virulent and non-virulent strains were statistically significant (*P*<0.01).

### Spleen-derived macrophages have exceptional phagocytic properties

Given the key role of splenic macrophages in pneumococcal clearance, we evaluated the capacity of splenic macrophages to internalise pneumococci. Splenic BALB/c macrophages were grown as primary cell cultures, washed and re-cultured for seven days in M-CSF supplemented medium. Cytoflourimetric data showed expression of the characteristic markers of splenic macrophages, CD11b, CD11c, F4/80 and SIGLEC-1 (Figure 2 H) [34]. Adhesion was evaluated by counting pneumococci after 45 min and phagocytosed bacteria were enumerated by plating after a further 30 min of antibiotic treatment (viable intracellular bacteria). Despite similar values in adherent cells (Figure 2 F), our data show higher numbers of intracellular bacteria for the rough DP1004 and for the G54 strain and less for the virulent D39 and TIGR4 (Figure 2 G). Essentially identical data where obtained when performing the experiment with splenic macrophages from C57BL/6 mice, while in contrast bone marrow macrophages from BALB/c mice and RAW264.7 macrophages showed different patterns of surface markers expression to the spleen macrophages and their phagocytosis of pneumococci showed no correlation to the extent of early clearance in the host (Figure S4). These data emphasise the importance in the choice of cell lines for performing phagocytosis assays in vitro to assess pneumococcal clearance in vivo.

### Whole genome sequencing identifies genetic evidence for a single cell bottleneck and adaptive mutations

Pneumococci grown from the blood of 6 mice were subjected to whole genome sequencing (Table 3). In each case, the isolates had the identical antibiotic resistance phenotype and were therefore presumptively monoclonal. Two of the blood cultures were obtained from the same mouse, but at 24 or 48 h respectively, (mouse 3.1.5; Table 3). To identify possible mutations characterising the founding cell of the monoclonal blood culture, we searched for single nucleotide polymorphisms (SNP) present in all cells isolated from a given blood culture. Such a SNP would demonstrate that the re-expanded population arose from a single cell. We identified one or two SNPs in 100% of the bacterial populations from four out of six mice (3.1.5, 4.1.4, 4.2.2 and 4.2.6), when compared to bacteria from the challenge inoculum (Table 3). The identification of a SNP common to all bacteria of a given sample is conclusive genetic evidence that the pneumococcal populations were monoclonal. Further, the SNPs were either inter-genic, silent or in regions not predicted to be functional in pathogenesis. Thus, we conclude that these mutations were unlikely to be associated with changes in within-host fitness. This argues strongly that bacteremia was founded as the result of a stochastic process rather than the selection of fitter variants. However, further analysis identified a second set of SNPs in pneumococci of 5 of 6 blood cultures. Crucially, this second set of SNPs were only found in a proportion of the bacteria obtained from mouse blood and therefore must have occurred after the bottleneck. Further, these SNPs differed between isolates of different mice, but all were located within distinct sub-units of the pneumococcal F1/F0 ATPase operon (Table 3). In three bloods, more than one SNP was detected. To determine if more than one SNP in the ATPase operon occurred in a single cell, we sequenced single colony isolates of these populations. In all cases, where a multi-SNP profile would have been possible according to the genomic data, only clones with a single SNP within the F1/F0 ATPase operon were recovered. These isolates included FP490, a 3.2.4 derivative with a SNP in *atpA*, FP487, a 3.1.5 derivative with a SNP in *atpC*, and FP489 and FP498, two 4.1.6 derivatives with different SNPs in *atpD* (Table 1 and 3). In few cases subpopulations with mutations in other genes were detected (pilus sortase, potassium uptake protein, *metE*, and SP0760) (Table 3), but no confirmation by direct sequencing was performed for these genomic data and we do not think that these mutations are of major biological relevance.

**Table 3:** Genome sequencing data of pneumococcal colonies from monoclonal blood cultures.

Mouse ID	Challenge	SNP^a^	Amino acid		Gene mutated	% of population	Clone
3.2.4 48 h	FP321	G1418898T	A490E	SP1510	*atpA* F1F0 ATPase, F1 alpha	46	FP503
		G1421811C	P111A	SP1513	*atpB* F1F0 ATPase, F0 A	12	FP490
4.1.6 48 h	FP321	C0445820A	S10stop	SP0467	pilus sortase	15	
		G0460918C	I341M	SP0480	*trkA* potassium uptake protein	13	
		G1416894A	R330C	SP1508	*atpD* F1F0 ATPase, F1 beta	42	FP489
		C1417244T	G213D	SP1508	*atpD* F1F0 ATPase, F1 beta	12	FP498
		G1419354A	T338I	SP1510	*atpA* F1F0 ATPase, F1 alpha	17	FP504
4.2.6 48 h	FP321	G1418268T	P193Q	SP1509	*atpG* F1F0 ATPase, F1 gamma	29	FP506
		C1418341A	D169Y	SP1509	*atpG* F1F0 ATPase, F1 gamma	12	
		G1420251T	A39E	SP1510	*atpA* F1F0 ATPase, F1 alpha	19	
		C1570673T	silent	SP1670	*murF* D-ala-D-alanine ligase	100	
4.1.4 24 h	FP122	G1488683T	T11K	SP1583	*pcnA* nicotinamidase	100	
3.1.5 24 h	FP318	T0248016G	intergenic	-	intergenic (*rpsG* SP0272)	100	
		C0555356A	L420I	SP0585	*metE* methionine synthase	61	
		AAAT1416121-	L115 fs^b^	SP1507	*atpC* F1F0 ATPase, F1 epsilon	58	FP487
3.1.5 48 h	FP318	T0248016G	intergenic	-	intergenic (*rpsG* SP0272)	100	
		C0555356A	L420I	SP0585	*metE* methionine synthase	65	
		C0719460A	N129K	SP0760	Hypothetical	12	
		G0804415T	intergenic	-	intergenic (*ilvE* SP0857)	14	
		AAAT1416121-	L115 fs^b^	SP1507	*atpC* F1F0 ATPase, F1 epsilon	62	
4.2.2 48 h	FP318	C1416893A	R330L	SP1508	*atpD* F1F0 ATPase, F1 beta	95	FP505
		C1162082T	R436H	SP1229	*fhs* tetrahydrofolate ligase	100	
		C0239125A	H310N	SP0265	glycosyl hydrolase, family 1	100	

a numbering refers to position in the TIGR4 genome (NC_003028).

b frame shift. All F1/F0 ATPase mutations were deposited in GenBank (accession KF705516 to KF705525).

### *Ex vivo* pneumococci with ATPase mutations show variation in metabolic fitness

**Figure 4:**
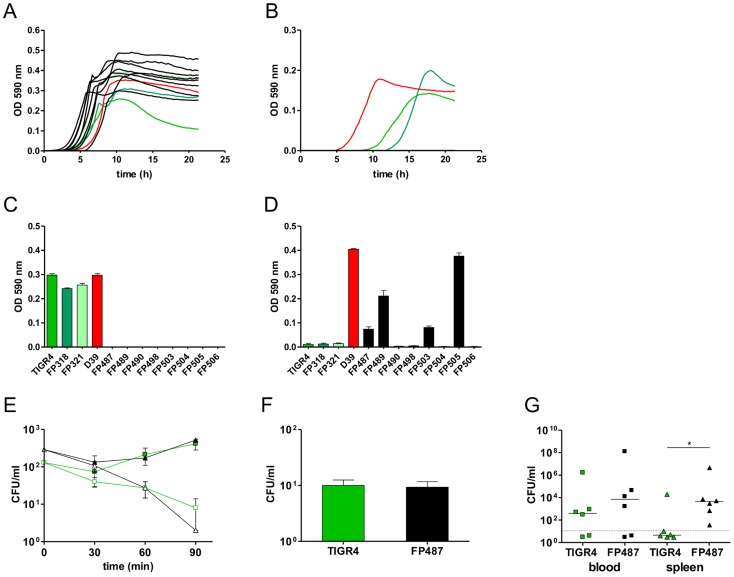
Phenotypes of single-colony blood culture isolates. Growth profiles of wild type strains (coloured lines) and ATPase mutants (black lines) in standard laboratory media THY (A) and TSB (B). D39 is indicated in red while TIGR4, FP318 and FP321 in different shade of green. (C, D) Maximum OD_590_ reached by wild type and ATPase mutants during growth with 80 mM K_2_HPO_4_ and pH 8.0 (D) and growth at pH 6.6 (E). Kinetics of opsono-phagocytosis assays in rotating blood with anti-type 4 serum (open symbols) and without antibodies (filled symbols) of parental strains (green) compared to a SP1507 *atpC* mutant (black). (F) Phagocytosis with primary cultures of spleen macrophages of TIGR4 and FP487 *atpC* mutant. (G) Bacterial counts in blood (squares) and spleen (triangles) at 72 h after i.v. infection of BALB/c mice (n = 6) with TIGR4 (green) and FP487 carrying a frame shifted SP1507 *atpC* gene (black). Data points below the cut off are negative. All data were analyzed by Student's *t*-test (*P*<0.05).

Phenotypic analysis of eight independent ex vivo blood isolates each having a mutation (SNP) in the ATPase (Table 1 and 3), showed normal colony morphology on agar plates and no significant change in their susceptibility to optochin. In liquid culture, the mutants showed normal or more efficient growth in Todd Hewitt Yeast Extract (Figure 4 A), but were unable to grow in other media (Tryptic Soy Broth) (Figure 4 B). Given that the F1/F0 ATPase is involved in multiple aspects of proton trafficking, we investigated the impact of pH, buffer composition and salt concentration on bacterial growth. Using a phenotype microarray for osmotic susceptibility using Biolog microtiter plates PM9, we compared the phenotype of parental strains derived from strain TIGR4 to the ex vivo mutants. The mutants had acquired a series of metabolic characteristics, also shared by strain D39 (Figure S5). Growth experiments performed in serial buffer and salt dilutions showed that TIGR4 and its isogenic derivatives used in the challenge experiments had a restricted pH optimum when compared to D39, which limited growth at potassium phosphate concentrations below 10 mM and pH below 6.8 (Figure 4 D). Interestingly many of the mutants had gained this capacity, making them equally able to grow at low pH as D39. In contrast, high buffer concentrations (80 mM K_2_HPO_4_ and pH 8), inhibited growth of all mutants (Figure 4 C).

To investigate effects on intracellular pH homeostasis of the ATPase mutations we transformed the frame-shift in the *atpC* gene into the non-encapsulated strain DP1004. Using in vivo NMR, the *atpC* mutant and its parental strain were both shown to have an identical intracellular pH of 6.52 to 6.56 during active metabolism of glucose. No differences in susceptibility to neutrophil killing were observed when mutants were assayed in an opsonophagocytosis assay in the presence of type specific antibodies (Figure 4 E). Also, data of macrophage phagocytosis were unaltered in primary cultures of splenic macrophages (Figure 4 F). To check for any fitness cost *in vivo*, the encapsulated *atpC* mutant was compared to the challenge strain FP321 in our i.v. mouse sepsis model. At early time points both strains showed comparable blood counts (data not shown). Also at 72 h post-challenge, bacterial counts in blood were similar, but bacterial spleen counts for the *atpC* mutant were significantly increased when compared to the wild-type (Figure 4 G).

## Discussion

We have investigated the pathogenesis of pneumococcal bacteraemia following intravenous inoculation of mice with three isogenic clones (variants). In our model, the infection followed the classic, three phase pattern in which a majority of pneumococci are cleared in the first minutes post-challenge. This leads to an “eclipse phase” of several hours in which bacterial numbers decline further or are undetectable. This is followed by the emergence of sustained and high density bacteraemia in a proportion of the challenged animals [8], [10]. By analysing the survival in the blood of three isogenic variants of *S. pneumoniae*, we observed that the majority of blood cultures arose from only one of the three variants. We used a statistical model to characterise the infection dynamics in which the number of bacteria starting the infection in each invasion event is w and the number of times this happens is k [23]. From the model, we could infer that the number of bacteria at the origin of infection is below 2 (w = 1). Thus, it follows that bacteraemia was generated by either (a) a single bacterium establishing a population in the blood in a single invasion event or (b) several bacteria each of which independently established a population in distinct invasion events. The probability of (b) is small (about 5% in our data, because the probability of two or more invasion events occurring is about 5%). Genome sequencing provided genetic evidence in 4/6 cases that monoclonal bacteraemia did actually start from a single bacterial cell (w = 1) confirming the first statement. For the remaining 2/6 cases we could not determine w = 1 by genome sequencing as we could not distinguish several invasions of a single bacterial variant from one invasion of several cells of the same variant without any SNPs. More complex is the experimental observation of invasive events. For this we could document bacteraemia in mice with previous negative blood samples (k≥1) and in other mice the increase of variants in serial blood samples (k>1). Since after the first 24 h the observed numbers of both these types of invasion events are similar, this strongly favours the occurrence of polyclonal infections resulting from independent, not cooperative action. In the case of *H. influenzae* it had been hypothesised that the single cells giving rise to the monoclonal infection might be selected by within-host evolution [23]. Our work now tests this hypothesis by whole genome sequencing. The data show in two cases absence of any SNPs and in four cases SNPs that apparently do not indicate selection for virulence. Despite the low numbers, it suggests that the single cells at the origin of infection apparently have no advantage (higher virulence) over the other cells in the population. Such results show that the bacteria in the challenge dose act independently to give rise to infection, that each has a similar probability of causing infection and that a dose near the LD50, a single cell may initiate disease. These criteria satisfy the theory of independent action [16], [17], [22], [23]. As such our investigation provides strong evidence that the single founding cell of an invasive infection is the result of a stochastic event. However, it must be emphasised that epigenetic variations would have eluded our genetic and genomic analysis.

Previously published studies have shown a major role for splenic macrophages in the initial clearance of pneumococci. In the seminal investigations of Brown et al. [35], they conclude: “… it appears that an anatomically normal spleen plays a unique role in the clearance of experimental pneumococcal bacteraemia, and that this role is of increasing importance as the pathogenicity of the invading organism increases”. Our data provide evidence that splenic macrophages have properties not found in those derived from other tissue sites, with respect to their efficiency to ingest and kill pneumococci. It is worth noting that the impressive efficiency of splenic clearance *in vivo* in the non-immune host is somewhat at odds with the relatively inefficient ingestion and killing of pneumococci in standard *in vitro* phagocytosis assays [34]. The innate host factors that result in the removal of the vast majority of bacteria within 45 minutes of challenge [11], [35], [36] deserve further attention.

Despite the efficiency of splenic macrophages in clearance, sustained bacteraemia eventually occurs after an eclipse phase of several hours during which bacteria are largely undetectable in blood. Similar data were obtained in work based on intranasal inoculation of *H. influenzae*, where also mixed blood cultures were detected in the first minutes after challenge and before the eclipse period [25]. We propose for our intra venous injection model that during this time, a fraction of the inoculated bacteria are sequestered in extravascular tissues, most probably in the spleen, in accordance with our data on positivity of bacterial spleen counts also in mice with negative blood cultures (Figure S1). This emergence of a clone from the potential splenic focus into the “sterile” bloodstream can be viewed as equivalent to the “invasive events” described for models which consider more than one organ system [16], [18], [21], [23]–[25]. Sustained bacteraemia is initiated from replication of one bacterial cell, perhaps a stochastic event in which the first replicon to reach a threshold biomass sufficient to seed the blood “wins the day”. The exponential increase in the number of bacteria in the blood is consistent with contributions from both intravascular and extravascular replication of pneumococci. We favour a scenario in which, at a challenge dose below the LD50, the rate of replication occurring in the extra-vascular site, followed by seeding of bacteria to the blood, exceeds host clearance rates thereby resulting in progressively more severe bacteraemia. Our data do not infer that only one pneumoccocus survives the initial host clearance, but rather that from those that do survive; only one cell initiates bacteraemia. The observed increase of polyclonal infections over time, as predicted also by the independent action hypothesis, is in accordance with the doubling of the ratio of infected mice at those time points (0.31 at 24 h to 0.67 at 72 h) [22]. The strong positive selection which drives the emergence of the ATPase-SNP subclones during the later phase of the infection is novel with respect to previous models (i.e. the live-death model), which postulates a neutral selection during this phase [16], [17], [37]. The in depth genomic analysis, in contrast to previous works [16], [17], [37], shows evidence for a more dynamic behaviour of the infecting bacterial population with an increase in heterogeneity of the monoclonal population over time due to a strong positive selection after the single cell bottleneck.

However, we observed added complexity; the residual, but inadequate, innate clearance mechanisms exert a selective pressure resulting in the emergence of adaptive mutants. Sequencing of bacteria from blood revealed that in most mice the bacterial clones had each acquired SNPs in different sub-units of the pneumococcal F1/F0 ATPase gene. This apparently high frequency of mutations, given the relatively small biomass of pneumococci in each animal, is consistent with the estimated mutation rates of up to 5×10^−4^ per genome described recently for pneumococci during one-cell bottleneck *in vitro* passages [38]. The selection for altered function of the ATPase, was found only in a proportion of the bacteria making up the population obtained from blood, compelling evidence that the ATPase mutations must have occurred after the single cell bottleneck. As stated above, the observation of subclones being selected during the bacteremic phase underlines a highly dynamic situation, which extends over the neutral two stage infection models [16], [17], [37].

In pneumococci, it has been recognised that ATPase mutations occur at high frequency during pneumococcal infection in humans, possibly in response to oxidative stress [39], and have been described both in vitro and in clinical isolates [40]–[44]. Polymorphisms in F0_atpA and F0_atpC (the trans-membrane part of the ATPase) were found to confer phenotypes of reduced susceptibility to optochin, quinine and mefloquine [40], [42], [43]. In particular, the detection of optochin resistant pneumococci in clinical samples is well described [44], as it has a practical impact on pneumococcal identification in the diagnostic laboratory [41]. None of the *ex vivo* ATPase mutants in our investigation were optochin resistant and the SNPs accordingly did not map to the optochin resistance conferring regions.

The F1/F0 ATPase is encoded by a highly conserved eight-gene operon and, as in aero-tolerant anaerobes, it is involved in the maintenance of intracellular pH through the generation of a membrane proton gradient [45]. In some of the mutants we were able to identify a clear metabolic benefit of the mutations which enabled growth at pH lower than 6.8, albeit all mutants showed that loss of capacity to grow at pH above 7.8. Interestingly the phenotype of TIGR4 mutants recovered from blood was not different from other virulent pneumococcal stains, such as D39. The high frequency of mutation observed here, given by the many different sites mutated, strongly suggests within-host adaptation through selective pressure during sepsis. While *in vitro* susceptibility of the ATPase mutants to antibody mediated neutrophil killing and macrophage phagocytosis was essentially unaltered, the phenotypic consequence of the ATPase mutations may be linked to a gain in fitness related to the increased survival of bacteria within the splenic, extravascular focus that provides the source of pneumococci re-seeding the blood and sustaining the progressively escalating and ultimately lethal bacteraemia. In agreement with this hypothesis is the recent description of inhibition of the own F1F0 ATPase by both *Salmonella enterica* and *Mycobacterium tuberculosis* as strategy to withstand phagolysosomal activity [46].

In summary, we propose that after the majority of the bacteria of the challenge inoculum have been removed, a few bacteria survive the predominantly lethal activity of splenic macrophages and neutrophils. From these rare survivors, single pneumococcal cells may start to replicate and initiate seeding of the blood resulting in a steady state bacteraemia in which efficient host clearance is off-set by re-seeding from the original, persisting extravascular reservoir of bacteria. These extravascular bacteria are subjected to strong selection for adaptive mutations. Later during infection, selected subpopulations of the initial clone may become part of the bacterial population causing disease. These observations are in accordance with a two stage model of infection where independent action generating the initial stochastic event is followed by a dynamic birth-death phase which increases heterogeneity due to strong selection [16], [17], [24]. In the case of the model organism *S. pneumoniae*, our data show different selective pressures shaping the invasive bacterial population during different phases of infection [16]. Given the demonstration that pneumococci are independent in generating disease in our rodent model and that less than twenty per cent of human pneumonia cases are bacteraemic [3], we hypothesize that human pneumococcal bacteraemia is generally monoclonal originating from a single cell in analogy to the monoclonal meningitis case recently described [47]. Presentation of a model which foresees development of invasive disease from a single bacterium and strong selection during outgrowth represents an important example on which to model fitness selection during invasive infection.

## Materials and Methods

### Pneumococcal strains and growth conditions

Three isogenic *zmpC* knock-out mutants of TIGR4 (FP122, FP318 and FP321) that differed only for the resistance marker, *ermB* (erythromycin resistance), *aad9* (spectinomycin resistance) and *aphIII* (kanamycin resistance), respectively were constructed for co-infection studies with isogenic clones [28], [48]. The experiments with mice depleted of macrophages and neutrophils were done with four different strains: TIGR4 (serotype 4; strain FP321 *zmpC::aphIII*), G54 (serotype 19F; erythromycin and tetracycline resistant) [49], [50], D39 (serotype 2; strain FP335 *bglA::aad9*; gift of Hasan Yesilkaya, Leicester), and the streptomycin resistant non-encapsulated D39 derivative DP1004 [51], [52]. The transfer of the *atpC* frame-shift into DP1004 was performed by transformation of a marker flanked by two PCR fragments, one of which containing the frame-shift. This was possible since the *atpC* SNP is only 76 bp from the end of the operon. Two representative transformants FP499 and FP500 were confirmed by sequencing. The series of ATPase mutants isolated are described in Table 3, while all other strains are listed in Table 1. Strains were cultured in Tryptic Soy Broth (TSB, Liophilchem, Teramo) or Todd Hewitt (THY, Oxoid, Milano) supplemented with 0.5% Yeast Extract (Liophilchem). Solid media were blood agar plates (Tryptic soy agar, Difco) supplemented with 3% horse blood (Biotech, Grosseto). The colony morphology was checked on Todd-Hewitt agar plates containing 200 units/ml of catalase (Sigma-Aldrich, Milano, Italy) [53], [54]. Antibiotics were used at the following concentrations: 1 mg/L erythromycin, 500 mg/L kanamycin, 100 mg/L spectinomycin and 500 mg/L streptomycin (all from Sigma-Aldrich).

### Phenotypic assays of *atpC*

The intracellular pH was determined by Nuclear magnetic resonance (NMR). In brief, 400 ml of mid log pneumococcal cells grown in Todd Hewitt broth were pelleted and mixed with 1 ml of sodium alginate 6% (w/v 0.9‰ NaCl). Mixture were extruded manually through 25G needle on a surface of 0.25 M CaCl_2_ solution. The small drops were washed and transferred in the 10 mm NMR tube. NMR ^31^P spectra were recorded on a Bruker DRX 600 instrument operating at 242.9 MHz. ^31^P spectra were recorded with a 1.5 s repetition time and 45°flip angle. Line broadening of 10 Hz were applied before Fourier Transform. ^31^P chemical shift were determined by comparison with external standard Trisodium trimetaphospate at −20.80 ppm. Intracellular pH was determinate by P_i_ (intracellular phosphate) chemical shift in phosphate-free perfusion model [55]. Active metabolism of pneumococci was confirmed by acidification of the extracellular medium during the experiment carried out at 37°C.

Growth profiles of wild type strains and ATPase mutants were assayed both in standard laboratory media and in defined media. Standard laboratory media included TSB (Liophilchem) and Todd-Hewitt broth supplemented with Yeast Extract (0.5%) (Oxoid). Defined media were prepared in CAT medium by adding serial concentration of potassium phosphate buffer with different range of pH (6 to 8) and by adding several concentration of K_2_HPO_4_ as source of salt. CAT medium was composed by: Casitone (10gr/l) (Becton Dickinson), Tryptone (10 gr/l) (Oxoid), Yeast Extract (1 gr/l) (Liophilchem), NaCl (5 gr/l) (Panreac, Milano, Italy), Catalase (200 U/ml) (Sigma-Aldrich) and Glucose (0.2%) (J.T. Baker, Milano, Italy). Metabolism of pneumococcal strains including wild type and ATPase mutants were assayed by Phenotype MicroArray (PM) microplate PM9 containing a total of 96 different osmolyte sources. PM technology measures active metabolism by recording the irreversible reduction of tetrazolium violet to formazan as an indirect evidence for NADH production. PM procedures were carried out as previously described (Viti C 2009). Quantitative colour change were recorded automatically every 15 min for a period of 72 h. Analyses were performed by the Omnilog-PM Software (Biolog, inc.) and data were filtered using average height as a parameter.

### Ethics statement

Animal experimentation in Italy is regulated by Decreto Legislativo 116/92 and Directive 210/63/EU. The animal protocol was approved by the “Comitato Etico Locale” of the Azienda Universitaria Ospedaliera Senese and received thereafter the relative project licence issued by the Italian Ministry of Health (193/2008-B).

### Mice

Six to seven-weeks old female CD1, BALB/c, and CBA/Ca mice were purchased from Charles River Italia (Lecco, Italy). For the bottleneck experiments, outbred CD1 mice were used, while BALB/c mice were chosen for both *in vivo* macrophage and neutrophil depletion and *ex vivo* experiments. CBA/Ca data are shown only for comparison of the dynamics of the early phases of infection. Animals were sacrificed by intraperitoneal (i.p.) injection of xylazine hydrochloride and zolazepam tiletamine cocktail (Xilor 2%, Bio 98 S.r.l., Bologna, Italy and Zoletil 20, Virbac S.r.l., Milano, Italy). Mice were kept at the animal facility of the LAMMB, University of Siena, according to its guidelines for the maintenance of laboratory animals [48], [56]–[58]. Blood samples from mice were collected by sub-mandibular vein or cardiac puncture under terminal anaesthesia. To prevent blood coagulation, 100 U/ml of heparin (MS Pharma, Milano, Italy) was added. All the collected organs (spleen, lung, liver and kidney) were homogenized in 1 ml of TSB, and then frozen at −80°C after making to 10% v/v of glycerol.

### Challenge experiments

Two series of experiments were performed in order to define the bottleneck for invasive pneumococcal infection with a total of 68 CD1 mice. Mice were challenged intravenously (i.v.) as described [48], [56]–[58] with a mixture of the three isogenic TIGR4 derivatives (FP122, FP318 and FP321) at 3.3×10^5^ CFU each/mouse. At pre-set time points blood samples were collected and selected groups were sacrificed for obtainment of spleen samples. Two blood samples from each animal, taken at different time points, are reported in Figure 1. Bacteria were enumerated by plating on selective media. The dose of the experiment was decided after having observed in a preliminary experiment 5/8 mixed and 3/8 monoclonal infections using two pneumococcal clones at a dose of 2×10∧6 (data not shown).

A pilot experiment for comparison of virulence in CD1, BALB/c and CBA/Ca mice was carried out by infecting i.v. four mice each with a mixture of G54, D39 and TIGR4 (3×10^5^ CFU each/mouse). Three blood samples per mouse were obtained.

For depletion of macrophages, BALB/c mice were treated 24 h prior to challenge by i.p. injection with 750 µl of a suspension of clodronate (CL_2_MBP) liposomes. One control group received PBS-containing liposomes [31] and the other was untreated. Clodronate was encapsulated in liposomes, as described earlier [31] and was a gift of Roche Diagnostics (Mannheim, Germany). Neutrophil depletion was performed by a single i.p. injection of 150 µg/mouse of anti-GR-1 antibody (Ly6G and Ly6C, clone RB8-8C5; Becton Dickinson) 24 h prior to infection [32], [33]. Two control groups were either left untreated or administered with a rat isotype control antibody IgG2b K (kappa) (Becton Dickinson). Groups of mice depleted of macrophages or neutrophils were infected i.v. with 1×10^6^ CFU/mouse containing 2.5×10^5^ CFU of each TIGR4, D39, DP1004 and G54. Bacterial viable counts were determined at preset time points.

The virulence of the ATPase mutant FP487 (*atpC* mutant) was assayed in parallel with TIGR4. BALB/c mice (n = 6) were infected with 1×10^6^ CFU/mouse i.v. and blood and spleen samples collected at 72 h.

### Macrophage phagocytosis

Spleen and bone marrow macrophages were isolated from mice using a modified protocol previously described [34]. Cells were cultured in medium supplemented with 25 ng/ml of recombinant M-CSF (Invitrogen) and re-seeded at day 7 at the concentration of 2×10^5^ cell/ml. After 24 h, 0.1 ml of pneumococci cultured to OD_590_ 0.25 were added. After 45 min plates were washed and reincubated with 10 mg/L of penicillin and 200 mg/L of gentamicin for 30 min. Intracellular bacteria were enumerated after lysis with saponin 1%. Phagocytosis of RAW264.7 macrophages followed the same protocol, but in addition samples were reincubated after removal of the antibiotics for an additional hour in fresh medium.

### Flow cytometry analysis

Flow cytometric analysis was conducted on bacteria suspended in 1% v/v paraformaldehyde in PBS on a FACScalibur machine (Becton Dickinson, California, USA). To verify macrophage and neutrophil depletion, homogenised organ samples were washed in DMEM (Sigma-Aldrich) and non-specific binding was blocked with FcR blocking agent [59]. Cells were incubated 30 min with 1 µg of specific fluorochrome-conjugated antibodies per 10^6^ cells. Neutrophils were stained using a rat anti-GR-1 antibody (MACS, Bologna, Italy). Macrophages were detected with rat anti-F4/80 mAb (BM8 clone; Abcam, Milano, Italy), and a rat anti-mouse CD11b mAb (Becton-Dickinson). Surface markers of macrophages were analysed using the following antibodies: anti-F4/80 mAb, anti-mouse CD11b mAb, anti-CD11c mAb (eBioscience), anti-mouse SIGNR1/CD209b Ab, goat IgG control Ab, anti-mouse Siglec-1 mAb, rat IgG2A Isotype control Ab, anti-mouse MARCO mAb and rat IgG1 isotype control Ab (R&D Systems).

To assay for the presence of anti-pneumococcal antibodies in mouse sera, the four pneumococcal strains TIGR4, G54, D39 and DP1004 were blocked in PBS-BSA 1% v/v for 30 min at 37°C and incubated for 1 h at 37°C with sera (1∶100) obtained from BALB/c mice and the positive anti-serotype 2 control serum (Staten Serum Institute, SSI, Copenhagen, DK). Samples were marked with anti-mouse IgG (1∶64) or anti-rabbit IgG (1∶160; Sigma-Aldrich).

### *Ex vivo* blood survival assay

In order to evaluate the capacity of whole blood to kill or inhibit the multiplication of pneumococci and to investigate the effect of specific antibodies, *ex vivo* experiments were set up. Blood from BALB/c mice was collected into tubes containing heparin and infected with pneumococci. For the assay of opsono-phagocytosis of ATPase mutants 1×10^4^ CFU/ml of parental and ATPase mutant were inoculated in blood and incubated in rotation. The anti-type 4 serum (SSI) was used at 1∶50 dilution. For the evaluation of growth of pneumococci in blood 3×10^5^ CFU/ml of G54, D39 and TIGR4 were inoculated in rotating blood. The efficacy of type 2 anti-serum (SSI) on D39 and its non-encapsulated derivative DP1004 was assayed as above using a inoculum of 3×10^5^ CFU/ml and a 1∶100 dilution of the serum.

### Whole genome sequencing

Chromosomal DNA was extracted using the High Pure PCR Template preparation kit (Roche). Whole genome sequencing was performed by the Institute of Applied Genomics and IGA Technology Services srl (University of Udine, Italy) using an Illumina (Solexa) Genome Analyzer II platform [60]. Reads of both, parent and mutant strains, were aligned to the reference genome of TIGR4 (accession NC_003028) using the Mosaik Assembler suite (The MarthLab, Boston College, Massachusetts, USA). Single nucleotide polymorphisms (SNPs), insertions and deletions (INDELs) were retrieved with VarScan software [61]. SNPs and INDELs of the challenge strains were subtracted from those found by aligning the blood isolates. All F1/F0 ATPase mutations were re-sequenced by the Sanger method and deposited in GenBank (accession KF705516 to KF705525).

### Statistical analysis

In order to evaluate the number of bacteria at the origin of blood infection, a model derived from that previously described [23], was developed. A full description of the statistical model is given in the supplementary materials. Statistical analysis of bacterial counts in blood and organs was performed by the Student's *t*-test for data reported in Figures 3, 4, S2 and S3. The analysis of different bacterial blood clearance at 5 and 30 min and the differences in bacterial phagocytosis and data of phenotype microarray were performed using Kruskal-Wallis and Dunn's multiple comparison post test (Figure 2 C–F and S5). Values of *P*<0.05 were considered statistically significant. The Fluorescence Index (Figure S3 K) was calculated by multiplying the percentage of positive events with the geometric mean fluorescence intensity (GeoMean).

## Supporting Information

Figure S1 **Paired spleen and blood counts of mice from** **Figure 1** **.** Blood (black) and spleen counts (white) of single mice (n = 12) at 24 h (A), 48 h (B) and 72 h (C) after i.v. challenge with a mixture of three isogenic *S. pneumoniae* TIGR4 variants (3×10^5^ CFU/each strain). Data are from a subset of mice shown in Figure 1.
(PDF)

Figure S2 **Bacterial counts in organs of BALB/c mice depleted of neutrophils or macrophages (the data for blood and spleen are those in** **Figure 3** **).** A mixture of four different pneumococcal strain (TIGR4, D39, DP1004 and G54) were injected i.v. in BALB/c mice at the challenge dose of 2.5×10^5^ CFU/each strain (1×10^6^ CFU/mouse). Bacterial counts in blood and organs over time are reported for untreated mice (A1, A2, A3 and A4), clodronate liposomes treated mice (B1, B2, B3 and B4) and anti-GR-1 mAb treated mice (C1, C2, C3 and C4). Blood cultures are shown with red lines, while CFU counts in the spleen, lung, liver and kidney are shown as blue, yellow, green and pink lines respectively. All samples were collected at different time points for 13 h, with the exception of mice treated with clodronate (8 h). Every organ was homogenized in 1 ml of medium and CFU/ml refers to counts for the whole organ. Data are reported as the mean ± SD of bacterial counts (n = 3–9).
(PDF)

Figure S3 **Control experiments for cell depletion** ***in vivo*** **, serum antibodies and phagocytosis in BALB/c mice.** BALB/c mice were infected by the i.v. route with a mixture of four different strains of *S. pneumoniae*: TIGR4 (green), D39 (fill red circles), DP1004 (open red circles) and G54 (blue) at the challenge dose of 2.5×10^5^ CFU/each strain (total 1×10^6^ CFU/mouse). Bacterial blood counts at different time point are reported for infected mice treated with clodronate liposomes (A), PBS liposomes (B), anti-GR1 mAb (E), isotype control rat IgG2b,k (F) and untreated mice (C and G). Data are represented as the mean ± SD of blood bacterial counts of three mice. Macrophage depletion (D) and neutrophil depletion (H) were confirmed by flow cytometry analysis with specific antibodies: anti-F4/80 and anti-CD11b for macrophage in the spleen and liver (D) and anti-GR-1 for neutrophil (H). Mean ± SD of triplicate of independent experiments are shown. (I) Growth of D39 (red), TIGR4 (green) and G54 (blue) pneumococcal strains in rotated fresh blood from BALB/c mice. A representative experiment is reported. (J) Effect of anti-capsular serotype 2 serum (1∶100) to survival of 3×10^5^ CFU/ml of D39 (red bar) and its non-encapsulated derivative DP1004 (open bar) in mouse blood incubated for 1 h. Mean ± SD of three independent experiments are reported and statistical analysis is performed by Student's *t*-test. (K) Binding of anti-type 2 specific antibody (1∶100) to whole pneumococci TIGR4 (green bar), D39 (red bar), G54 (blue bar) and rough DP1004 (red open bar) after 1 h incubation at 37°C. No binding was observed with non-immunized serum (1∶100) from na¿ve BALB/c mice. Data are represented as FI ± SEM of three independent experiments.
(PDF)

Figure S4 **Phagocytosis and surface marker characterization of different type of macrophages.** Adhesion (A) and invasion (phagocytosis) (B) of four different pneumococcal strains (G54, TIGR4, D39 and DP1004) in primary spleen macrophages (SPM) isolates from C57BL/6 mice. Each symbol indicates a single value and results are represented as mean ± SD (n = 3). (C) Cytofluorimetric analysis of surface markers of spleen macrophages from C57BL/6 mice. A representative experiment was reported. Invasion of different pneumococci (G54, TIGR4, D39 and DP1004) in bone marrow macrophages (BMM) isolates from BALB/c mice (D) and in RAW264.7 macrophage cell line (F). Data (n = 6–12) are reported as scatter plots to better evidence the number of negative assay. Dashed lines indicate the detection limits for positive samples. Relative analysis of surface marker expression of bone marrow macrophages (E) and RAW264.7 (G) by flow cytometry. Data are represented as per cent of positive cells of representative experiments.
(PDF)

Figure S5 **Phenotype MicroArray of pneumococcal strains.** Osmotic resistance of pneumococcal strains determined by Phenotype MicroArray. Bacterial metabolic activity determined in ethylene glycol (A), sodium nitrate (B) and sodium phosphate (C). Data were filtered using average height as a parameter (Biolog Omnilog-PM software). TIGR4 and the two TIGR4 derived challenge strains are in green, D39 in red and the mutants in black.
(PDF)

Table S1 **Distribution of TIGR4 variants in monoclonal blood cultures.**
(PDF)

Text S1 **Model to establish the number of invading bacteria.** Here we construct a statistical model to determine by which of two ways bacteraemia was generated.
(PDF)

## References

[1] Gray BM, Converse GM, Dillon HCJ (1980) Epidemiologic studies of *Streptococcus pneumoniae* in infants: acquisition, carriage, and infection during the first 24 months of life. J Infect Dis 142: 923–933. 7462701 10.1093/infdis/142.6.923

[2] Hogberg L, Geli P, Ringberg H, Melander E, Lipsitch M, et al (2007) Age- and serogroup-related differences in observed durations of nasopharyngeal carriage of penicillin-resistant pneumococci. J Clin Microbiol 45: 948–952. 17202280 10.1128/JCM.01913-06 PMC1829115

[3] Melegaro A, Edmunds WJ, Pebody R, Miller E, George R (2006) The current burden of pneumococcal disease in England and Wales. Journal of Infection 52: 37–48. 16368459 10.1016/j.jinf.2005.02.008

[4] Ispahani P, Slack RCB, Donald FE, Weston WC, Rutter N (2004) Twenty year surveillance of invasive pneumococcal disease in Nottingham: serogroups responsible and implications for immunisation. Arch Dis Child 89: 757–762. 15269078 10.1136/adc.2003.036921 PMC1720039

[5] Rudan I, Boschi-Pinto C, Mulholland K, Campbell H (2008) Epidemiology and ethiology of childhood pneumoniae. Bull World Health Organ 86: 408–416. 18545744 10.2471/BLT.07.048769 PMC2647437

[6] Kadioglu A, Weiser JN, Paton JC, Andrew PW (2008) The role of *Streptococcus pneumoniae* virulence factors in host respiratory colonization and disease. Nat Rev Microbiol 6: 288–301. 18340341 10.1038/nrmicro1871

[7] Chiavolini D, Pozzi G, Ricci S (2008) Animal models of *Streptococcus pneumoniae* disease. Clin Microbiol Rev 21: 666–685. 18854486 10.1128/CMR.00012-08 PMC2570153

[8] Wright DH (1927) Experimental pneumococcal septicaemia and anti-pneumococcal immunity. The Journal of Pathology and Bacteriology 30: 185–252.

[9] Rogers DE, Melly MA (1957) Studies on bacteriemia II. Further observations on the granulocytopenia induced by the intravenous injection of staphylococci. J Exp Med 105: 99–112. 13406171 10.1084/jem.105.2.99 PMC2136673

[10] Rogers DE (1960) Host mechanisms which act to remove bacteria from the blood stream. Bacteriol Rev 24: 50–66. 14438353 10.1128/br.24.1.50-66.1960 PMC441037

[11] Hosea SW, Brown EJ, Frank MM (1980) The critical role of complement in experimental pneumococcal sepsis. J Infect Dis 142: 903–909. 7462698 10.1093/infdis/142.6.903

[12] Hosea SW, Brown EJ, Hammer CH, Frank MM (1980) Role of complement activation in a model of adult respiratory distress syndrome. J Clin Invest 66: 375–382. 7400321 10.1172/JCI109866 PMC371720

[13] Van Wyck DB, Witte MH, Witte CL (1982) Synergism between the spleen and serum complement in experimental pneumococcemia. J Infect Dis 145: 514–519. 7069232 10.1093/infdis/145.4.514

[14] Brown EJ, Hosea SW, Frank MM (1983) The role of antibody and complement in the reticuloendothelial clearance of pneumococci from the bloodstream. Rev Infect Dis 5: 797–805. 10.1093/clinids/5.supplement_4.s797 6356294

[15] Levin BR, Antia R (2001) Why we don't get sick: the within-host population dynamics of bacterial infections. Science 292: 1112–1115. 11352067 10.1126/science.1058879

[16] Grant JA, Restif O, McKinley JT, Sheppard M, Maskell DJ, et al (2008) Modelling within-host spatiotemporal dynamics of invasive bacterial disease. PLOS Biology 6: 757–770. 10.1371/journal.pbio.0060074 PMC2288627 18399718

[17] Meynell GG, Maw J (1968) Evidence for a two-stage model of microbial infection. J Hyg (London) 66: 273–280. 4885483 10.1017/s0022172400041139 PMC2130631

[18] Sheppard M, Webb C, Heath F, Mallows V, Emilianus R, et al (2003) Dynamics of bacterial growth and distribution within the liver during Salmonella infection. Cell Microbiol 5: 593–600 296 [pii]. 12925129 10.1046/j.1462-5822.2003.00296.x

[19] Sacristan S, Malpica JM, Fraile A, Garcia-Arenal F (2003) Estimation of population bottlenecks during systemic movement of tobacco mosaic virus in tobacco plants. J Virol 77: 9906–9911. 12941900 10.1128/JVI.77.18.9906-9911.2003 PMC224588

[20] Barnes PD, Bergman MA, Mecsas J, Isberg RR (2006) Yersinia pseudotuberculosis disseminates directly from a replicating bacterial pool in the intestine. J Exp Med 203: 1591–1601. 16754724 10.1084/jem.20060905 PMC2118325

[21] Brown SP, Cornell SJ, Sheppard M, Grant AJ, Maskell DJ, et al (2006) Intracellular demography and the dynamics of Salmonella enterica infections. PLoS Biol 4: e349. 17048989 10.1371/journal.pbio.0040349 PMC1609125

[22] Meynell GG, Stocker BAD (1957) Some hypotheses on the aetiology of fatal infections in partially resistant hosts and their application to mice challenged with *Salmonella paratyphi-B* or *Salmonella typhimurium* by intraperitoneal injection. J Gen Microbiol 16: 58. 10.1099/00221287-16-1-38 13406218

[23] Margolis E, Levin BR (2007) Within-host evolution for the invasiveness of commensal bacteria: an experimental study of bacteremias resulting from *Haemophilus influenzae* nasal carriage. J Infect Dis 196: 1068–1075. 17763330 10.1086/520934

[24] Meynell GG (1957) The applicability of the hypothesis of indipendent action to fatal infections in mice given *Salmonella typhimurium* by mouth. J Gen Microbiol 16: 396–404. 13416517 10.1099/00221287-16-2-396

[25] Moxon ER, Murphy PA (1978) *Haemophilus influenzae* bacteremia and meningitis resulting from survival of a single organism. Proc Natl Acad Sci USA 75: 1534–1536. 306628 10.1073/pnas.75.3.1534 PMC411507

[26] Plunschke G, Mercer A, Kusecek B, Pohl A, Achtman M (1983) Induction of bacteremia in newborn rats *Escherichia coli* K1 is correlated with only certain O (lipopolysaccharide) antigen type. Infect Immun 39: 599–607. 6187683 10.1128/iai.39.2.599-608.1983 PMC347994

[27] Rubin LG (1987) Bacterial colonizzation and resulting from multiplication of a single organism. Reviews of Infectious Diseases 9: 488–493. 3299635 10.1093/clinids/9.3.488

[28] Oggioni MR, Memmi G, Maggi T, Chiavolini D, Iannelli F, et al (2003) Pneumococcal zinc metalloproteinase ZmpC cleaves human matrix metalloproteinase 9 and is a virulence factor in experimental pneumonia. Mol Microbiol 49: 795–805. 12864860 10.1046/j.1365-2958.2003.03596.x

[29] Weiser JN, Austrian R, Sreenivasan PK, Masure HR (1994) Phase variation in pneumococcal opacity: relationship between colonial morphology and nasopharyngeal colonization. Infect Immun 62: 2582–2589. 8188381 10.1128/iai.62.6.2582-2589.1994 PMC186548

[30] Ripoll VM, Kadioglu A, Cox R, Hume DA, Denny P (2010) Macrophages from BALB/c and CBA/Ca mice differ in their cellular responses to Streptococcus pneumoniae. J Leukoc Biol 87: 735–741. 20028774 10.1189/jlb.0509359

[31] van Rooijen N, Sanders A (1994) Liposome mediated depletion of macrophages:mechanism of action, preparation of liposomes and applications. J Immunol Methods 174: 83–93. 8083541 10.1016/0022-1759(94)90012-4

[32] Fleming TJ, Fleming ML, Malek TR (1993) Selective expression of Ly-6G on myeloid lineage cells in mouse bone marrow. RB6-8C5 mAb to granulocyte-differentiation antigen (Gr-1) detects members of the Ly-6 family. J Immunol 151: 2399–2408. 8360469

[33] Daley JM, Thomay AA, Connolly MD, Reichner SJ, Albina JE (2008) Use of Ly6G-specific monoclonal antibody to deplete neutrophils in mice. Journal of Leukocyte Biology 83: 64–70. 17884993 10.1189/jlb.0407247

[34] Alatery A, Basta S (2008) An efficient culture method for generating large quantities of mature mouse splenic macrophages. J Immunol Methods 338: 47–57. 18675819 10.1016/j.jim.2008.07.009

[35] Brown EJ, Hosea SW, Frank MM (1981) The role of complement in the localisation of pneumococci in the splenic reticuloendothelial system during experimental bacteremia. The Journal of Immunology 126: 2230–2234. 7229372

[36] Brown EJ, Hosea SW, Frank MM (1981) The role of the spleen in experimental pneumococcal bacteremia. J Clin Invest 67: 975–982. 7204580 10.1172/JCI110148 PMC370655

[37] Shortley G, Wilkins JR (1965) Independen-action and birth-death models in experimental microbiology. Bacteriol Rev 29: 102–141. 14295982 10.1128/br.29.1.102-141.1965 PMC441262

[38] stevens KE, Sebert ME (2011) Frequent beneficial mutations during single-colony serial transfer of *Streptococcus pneumoniae* . Plos Genet 7: e1002232. 21876679 10.1371/journal.pgen.1002232 PMC3158050

[39] Pericone CD, Bae D, Shchepetov M, McCool T, Weiser JN (2002) Short-sequence tandem and nontandem DNA repeats and endogenous hydrogen peroxide production contribute to genetic instability of Streptococcus pneumoniae. J Bacteriol 184: 4392–4399. 12142409 10.1128/JB.184.16.4392-4399.2002 PMC135236

[40] Munoz R, Garcia E, de la Campa AG (1996) Quinine specifically inhibits the proteolipid subunit of the F0F1 H+ -ATPase of *Streptococcus pneumoniae* . J Bacteriol 178: 2455–2458. 8636056 10.1128/jb.178.8.2455-2458.1996 PMC177963

[41] Pikis A, Campos JM, Rodriguez WJ, Keith JM (2001) Optochin resistance in *Streptococcus pneumoniae*: mechanism, significance, and clinical implications. J Infect Dis 184: 582–590. 11474432 10.1086/322803

[42] Martin-Galiano AJ, Gorgojo B, Kunin CM, de la Campa AG (2002) Mefloquine and new related compounds target the F(0) complex of the F(0)F(1) H(+)-ATPase of Streptococcus pneumoniae. Antimicrob Agents Chemother 46: 1680–1687. 12019076 10.1128/AAC.46.6.1680-1687.2002 PMC127268

[43] Dias CA, Agnes G, Frazzon AP, Kruger FD, d'Azevedo PA, et al (2007) Diversity of mutations in the atpC gene coding for the c Subunit of F0F1 ATPase in clinical isolates of optochin-resistant Streptococcus pneumoniae from Brazil. J Clin Microbiol 45: 3065–3067. 17626173 10.1128/JCM.00891-07 PMC2045260

[44] Nunes S, Sà-Leao R, De Lencastre H (2008) Optochin resistence among *Streptococcus pneumoniae* strains colonizing healthy children in Portugal. J Clin Microbiol 46: 321–324. 18032618 10.1128/JCM.02097-07 PMC2224255

[45] Krulwich TA, Sachs G, Padan E (2011) Molecular aspects of bacterial pH sensing and homeostasis. Nat Rev Microbiol 9: 330–343 nrmicro2549 [pii];10.1038/nrmicro2549 [doi] 21464825 10.1038/nrmicro2549 PMC3247762

[46] Lee EJ, Pontes MH, Groisman EA (2013) A bacterial virulence protein promotes pathogenicity by inhibiting the bacterium's own F1Fo ATP synthase. Cell 154: 146–156. 23827679 10.1016/j.cell.2013.06.004 PMC3736803

[47] Croucher NJ, Mitchell AM, Gould KA, Inverarity D, Barquist L, et al (2013) Dominant role of nucleotide substitution in the diversification of serotype 3 pneumococci over decades and during a single infection. PLoS Genet 9: e1003868. 24130509 10.1371/journal.pgen.1003868 PMC3794909

[48] Chiavolini D, Memmi G, Maggi T, Iannelli F, Pozzi G, et al (2003) The three extra-cellular zinc metalloproteinases of *Streptococcus pneumoniae* have a different impact on virulence in mice. BMC Microbiol 3: 14. 12841855 10.1186/1471-2180-3-14 PMC166150

[49] Pozzi G, Masala L, Iannelli F, Manganelli R, Havarstein LS, et al (1996) Competence for genetic transformation in encapsulated strains of *Streptococcus pneumoniae*: two allelic variants of the peptide pheromone. J Bacteriol 178: 6087–6090. 8830714 10.1128/jb.178.20.6087-6090.1996 PMC178474

[50] Dopazo J, Mendoza A, Herrero J, Caldara F, Humbert Y, et al (2001) Annotated draft genomic sequence from *Streptococcus pneumoniae* type 19F clinical isolate. Microb Drug Resist 7: 99–125. 11442348 10.1089/10766290152044995

[51] Pozzi G, Musmanno RA, Renzoni EA, Oggioni MR, Cusi MG (1988) Host-vector system for integration of recombinant DNA into chromosomes of transformable and nontransformable streptococci. J Bacteriol 170: 1969–1972. 2832394 10.1128/jb.170.4.1969-1972.1988 PMC211061

[52] Iannelli F, Chiavolini D, Ricci S, Oggioni MR, Pozzi G (2004) Pneumococcal surface protein C contributes to sepsis caused by *Streptococcus pneumoniae* in mice. Infect Immun 72: 3077–3080. 15102826 10.1128/IAI.72.5.3077-3080.2004 PMC387904

[53] Bidossi A, Mulas L, Decorosi F, Colomba L, Ricci S, et al (2012) A functional genomics approach to establish the complement of carbohydrate transporters in *Streptococcus pneumoniae* . PLoS ONE 7: e33320. 22428019 10.1371/journal.pone.0033320 PMC3302838

[54] Trappetti C, Ogunniyi AD, Oggioni MR, Paton JC (2011) Extracellular matrix fromation enhances the ability of *Streptococcus pneumoniae* to cause invasive disease. PLoS ONE 6: e19844. 21611130 10.1371/journal.pone.0019844 PMC3097209

[55] Ben-Horin H, Tassini M, Vivi A, Navon G, Kaplan O (1995) Mechanism of action of the antineoplastic drug lonidamine: 31P and 13C nuclear magnetic resonance studies. Cancer Res 55: 2814–2821. 7796408

[56] Kadioglu A, Cuppone AM, Trappetti C, List T, Spreafico A, et al (2011) Sex-based differences in susceptibility to respiratory and systemic pneumococcal disease in mice. J Infect Dis 204: 1971–1979. 22021621 10.1093/infdis/jir657

[57] Oggioni MR, Iannelli F, Ricci S, Chiavolini D, Parigi R, et al (2004) Antibacterial activity of a competence-stimulating peptide in experimental sepsis caused by *Streptococcus pneumoniae* . Antimicrob Agents Chemother 48: 4725–4732. 15561850 10.1128/AAC.48.12.4725-4732.2004 PMC529211

[58] Oggioni MR, Trappetti C, Kadioglu A, Cassone M, Iannelli F, et al (2006) Switch from planktonic to sessile life: a major event in pneumococcal pathogenesis. Mol Microbiol 61: 1196–1210. 16925554 10.1111/j.1365-2958.2006.05310.x PMC1618759

[59] Ciabattini A, Pettini E, Andersen P, Pozzi G, Medaglini D (2008) Primary activation of antigen-specific naive CD4^+^ and CD8^+^ T cells following intranasal vaccination with recombinant bacteria. Infect Immun 76: 5817–5825. 18838521 10.1128/IAI.00793-08 PMC2583588

[60] Zhou X, Ren L, Meng Q, Li Y, Yu Y, et al (2010) The next-generation sequencing technology and application. Protein Cell 1: 520–536. 21204006 10.1007/s13238-010-0065-3 PMC4875313

[61] Koboldt DC, Zhang Q, Larson DE, Shen D, McLellan MD, et al (2012) VarScan 2: somatic mutation and copy number alteration discovery in cancer by exome sequencing. Genome Res 22: 568–576. 22300766 10.1101/gr.129684.111 PMC3290792

[62] Lanie JA, Ng WL, Kazmierczak KM, Andrzejewski TM, Davidsen TM, et al (2007) Genome sequence of Avery's virulent serotype 2 strain D39 of *Streptococcus pneumoniae* and comparison with that of unencapsulated laboratory strain R6. J Bacteriol 189: 38–51. 17041037 10.1128/JB.01148-06 PMC1797212

[63] Tettelin H, Nelson KE, Paulsen IT, Eisen JA, Read TD, et al (2001) Complete genome sequence of a virulent isolate of *Streptococcus pneumoniae* . Science 293: 498–506. 11463916 10.1126/science.1061217

