# Supplementary material for: The Role of Host and Microbial Factors in the Pathogenesis of Pneumococcal Bacteraemia Arising from a Single Bacterial Cell Bottleneck
Source: PLoS Pathog. 2014 Mar 20;10(3):e1004026. doi: 10.1371/journal.ppat.1004026 (PMC3961388; doi:10.1371/journal.ppat.1004026)
Supplement: Figure S1 — Paired spleen and blood counts of mice from Figure 1 . Blood (black) and spleen counts (white) of single mice (n = 12) at 24 h (A), 48 h (B) and 72 h (C) after i.v. challenge with a mixture of three isogenic S. pneumoniae TIGR4 variants (3×10^5^ CFU/each strain). Data are from a subset of mice shown in Figure 1. (PDF) [file ppat.1004026.s001.pdf]

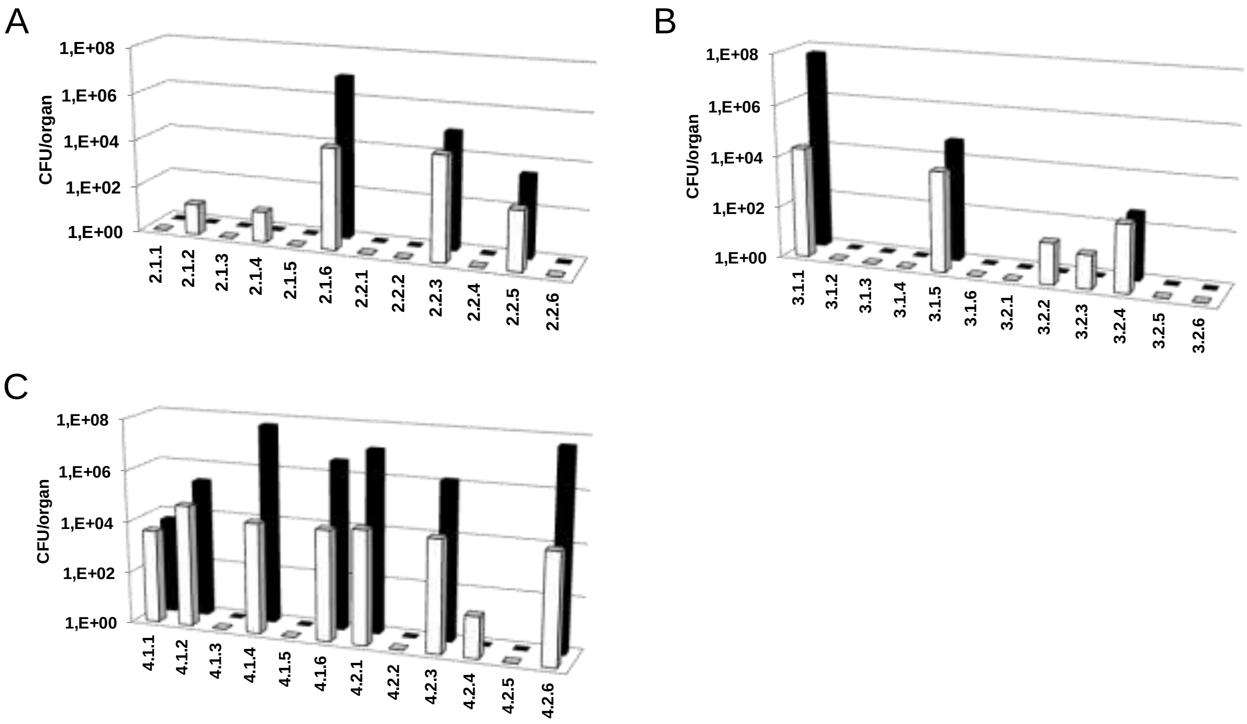

Figure S1. Paired spleen and blood counts of mice from Figure 1. Blood (black) and spleen counts (white) of single mice (n=12) at 24 h (A), 48 h (B) and 72 h (C) after i.v. challenge with a mixture of three isogenic *S. pneumoniae* TIGR4 variants (3×10^5 CFU/each strain). Data are from a subset of mice shown in Figure 1.
