# Supplementary material for: The Role of Host and Microbial Factors in the Pathogenesis of Pneumococcal Bacteraemia Arising from a Single Bacterial Cell Bottleneck
Source: PLoS Pathog. 2014 Mar 20;10(3):e1004026. doi: 10.1371/journal.ppat.1004026 (PMC3961388; doi:10.1371/journal.ppat.1004026)
Supplement: Figure S2 — Bacterial counts in organs of BALB/c mice depleted of neutrophils or macrophages (the data for blood and spleen are those in Figure 3 ). A mixture of four different pneumococcal strain (TIGR4, D39, DP1004 and G54) were injected i.v. in BALB/c mice at the challenge dose of 2.5×10^5^ CFU/each strain (1×10^6^ CFU/mouse). Bacterial counts in blood and organs over time are reported for untreated mice (A1, A2, A3 and A4), clodronate liposomes treated mice (B1, B2, B3 and B4) and anti-GR-1 mAb treated mice (C1, C2, C3 and C4). Blood cultures are shown with red lines, while CFU counts in the spleen, lung, liver and kidney are shown as blue, yellow, green and pink lines respectively. All samples were collected at different time points for 13 h, with the exception of mice treated with clodronate (8 h). Every organ was homogenized in 1 ml of medium and CFU/ml refers to counts for the whole organ. Data are reported as the mean ± SD of bacterial counts (n = 3–9). (PDF) [file ppat.1004026.s002.pdf]

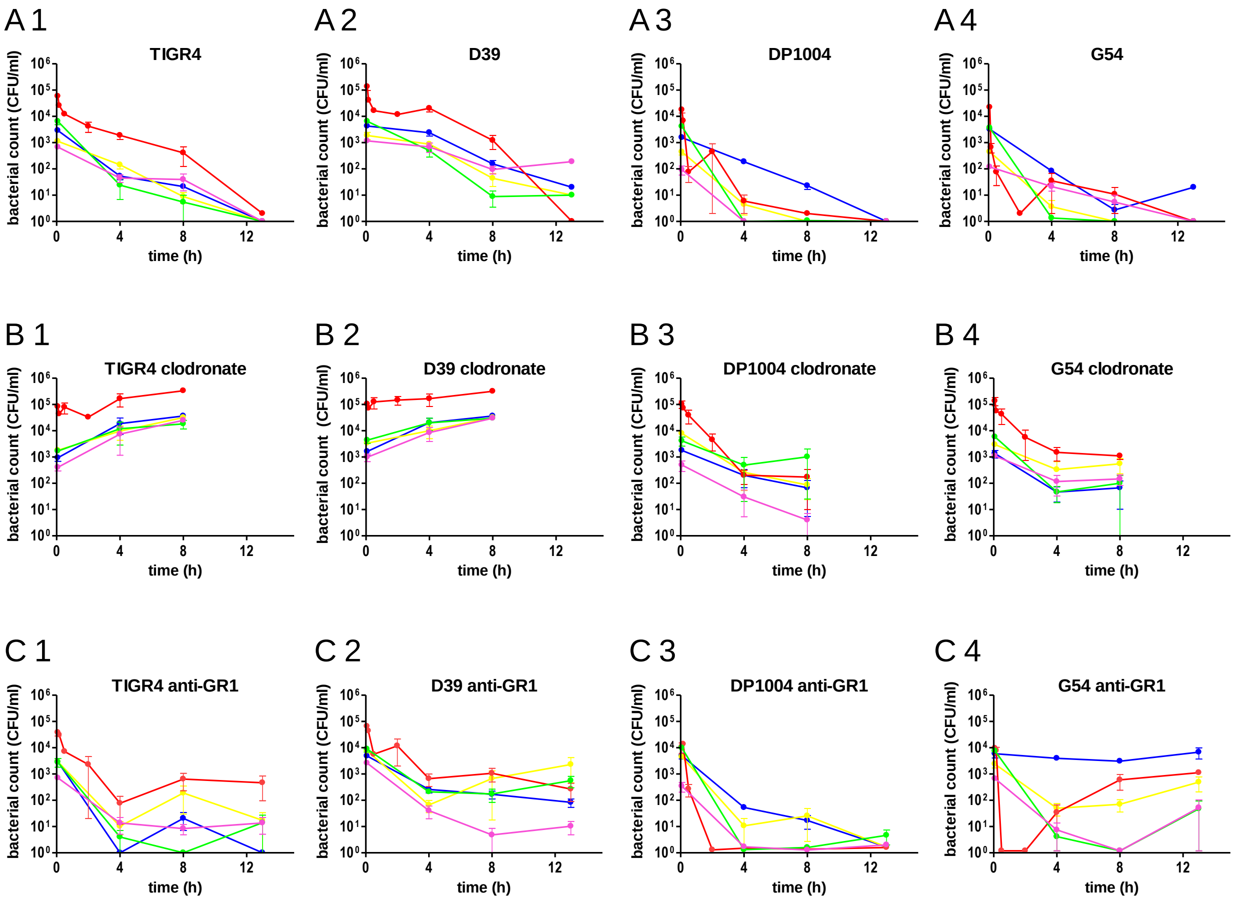

Figure S2. Bacterial counts in organs of BALB/c mice depleted of neutrophils or macrophages (the data for blood and spleen are those in Figure 3). A mixture of four different pneumococcal strain (TIGR4, D39, DP1004 and G54) were injected i.v. in BALB/c mice at the challenge dose of 2.5×10^{5} CFU/each strain (1×10^{6} CFU/mouse). Bacterial counts in blood and organs over time are reported for untreated mice (A1, A2, A3 and A4), clodronate liposomes treated mice (B1, B2, B3 and B4) and anti-GR-1 mAb treated mice (C1, C2, C3 and C4). Blood cultures are shown with red lines, while CFU counts in the spleen, lung, liver and kidney are shown as blue, yellow, green and pink lines respectively. All samples were collected at different time points for 13 h, with the exception of mice treated with clodronate (8 h). Every organ was homogenized in 1 ml of medium and CFU/ml refer to counts for the whole organ. Data are reported as the mean ± SD of bacterial counts (n=3-9).
