# Supplementary material for: The Role of Host and Microbial Factors in the Pathogenesis of Pneumococcal Bacteraemia Arising from a Single Bacterial Cell Bottleneck
Source: PLoS Pathog. 2014 Mar 20;10(3):e1004026. doi: 10.1371/journal.ppat.1004026 (PMC3961388; doi:10.1371/journal.ppat.1004026)
Supplement: Figure S3 — Control experiments for cell depletion in vivo , serum antibodies and phagocytosis in BALB/c mice. BALB/c mice were infected by the i.v. route with a mixture of four different strains of S. pneumoniae: TIGR4 (green), D39 (fill red circles), DP1004 (open red circles) and G54 (blue) at the challenge dose of 2.5×10^5^ CFU/each strain (total 1×10^6^ CFU/mouse). Bacterial blood counts at different time point are reported for infected mice treated with clodronate liposomes (A), PBS liposomes (B), anti-GR1 mAb (E), isotype control rat IgG2b,k (F) and untreated mice (C and G). Data are represented as the mean ± SD of blood bacterial counts of three mice. Macrophage depletion (D) and neutrophil depletion (H) were confirmed by flow cytometry analysis with specific antibodies: anti-F4/80 and anti-CD11b for macrophage in the spleen and liver (D) and anti-GR-1 for neutrophil (H). Mean ± SD of triplicate of independent experiments are shown. (I) Growth of D39 (red), TIGR4 (green) and G54 (blue) pneumococcal strains in rotated fresh blood from BALB/c mice. A representative experiment is reported. (J) Effect of anti-capsular serotype 2 serum (1∶100) to survival of 3×10^5^ CFU/ml of D39 (red bar) and its non-encapsulated derivative DP1004 (open bar) in mouse blood incubated for 1 h. Mean ± SD of three independent experiments are reported and statistical analysis is performed by Student's t-test. (K) Binding of anti-type 2 specific antibody (1∶100) to whole pneumococci TIGR4 (green bar), D39 (red bar), G54 (blue bar) and rough DP1004 (red open bar) after 1 h incubation at 37°C. No binding was observed with non-immunized serum (1∶100) from na¿ve BALB/c mice. Data are represented as FI ± SEM of three independent experiments. (PDF) [file ppat.1004026.s003.pdf]

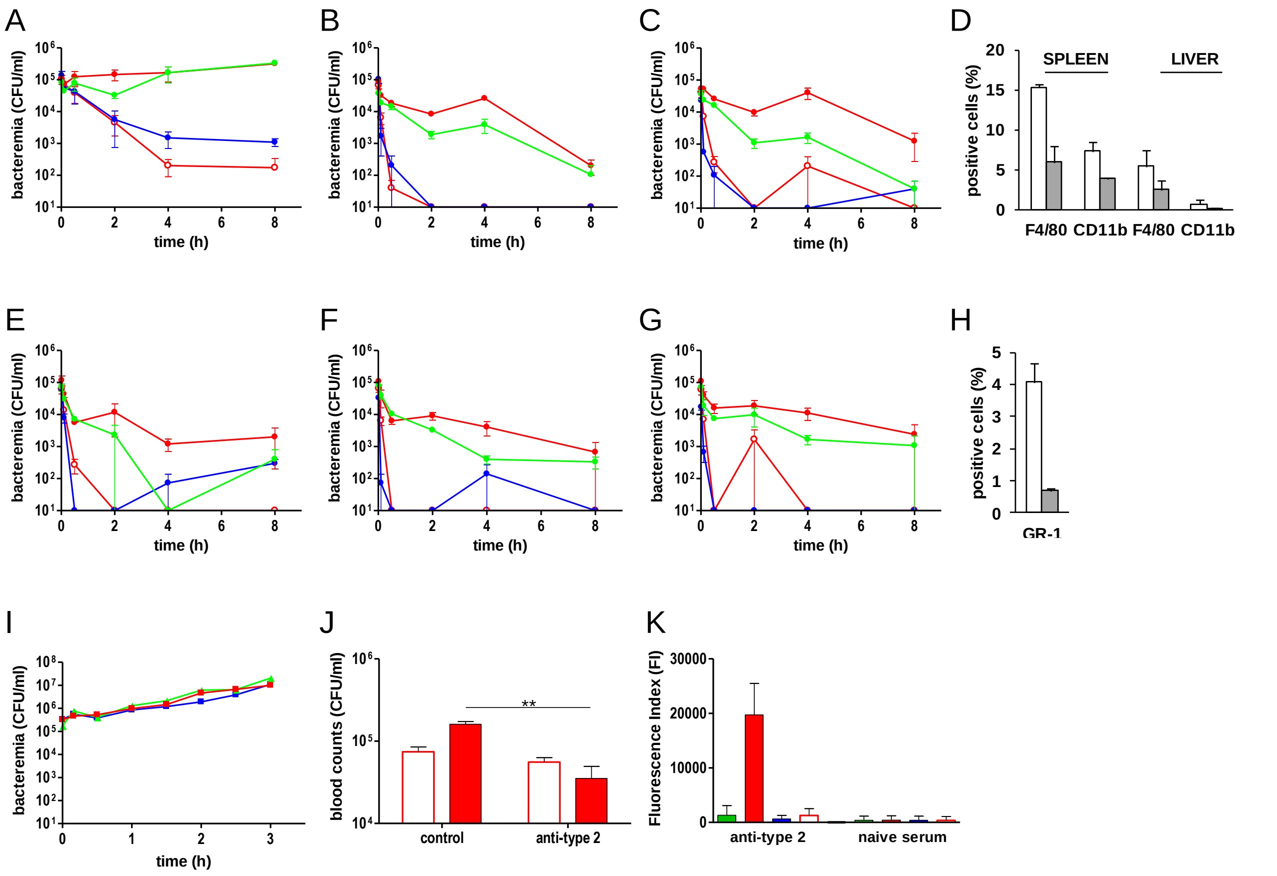

Figure S3. Control experiments for cell depletion *in vivo*, serum antibodies and phagocytosis in BALB/c mice. BALB/c mice were infected by the i.v. route with a mixture of four different strains of *S. pneumoniae*: TIGR4 (green), D39 (fill red circles), DP1004 (open red circles) and G54 (blue) at the challenge dose of 2.5×10^{5} CFU/each strain (total 1×10^{6} CFU/mouse). Bacterial blood counts at different time point are reported for infected mice treated with clodronate liposomes (A), PBS liposomes (B), anti-GR1 mAb (E), isotype control rat IgG2b,k (F) and untreated mice (C and G). Data are represented as the mean ± SD of blood bacterial counts of three mice. Macrophage depletion (D) and neutrophil depletion (H) were confirmed by flow cytometry analysis with specific antibodies: anti-F4/80 and anti-CD11b for macrophage in the spleen and liver (D) and anti-GR-1 for neutrophil (H). Mean ± SD of triplicate of independent experiments are shown. (I) Growth of D39 (red), TIGR4 (green) and G54 (blue) pneumococcal strains in rotated fresh blood from BALB/c mice. A representative experiments is reported. (J) Effect of anti-capsular serotype 2 serum (1:100) to survival of 3×10^{5} CFU/ml of D39 (red bar) and its non-encapsulated derivative DP1004 (open bar) in mouse blood incubated for 1 h. Mean ± SD of three independent experiments are reported and statistical analysis is performed by Student's *t*-test. (K) Binding of anti-type 2 specific antibody (1:100) to whole pneumococci TIGR4 (green bar), D39 (red bar), G54 (blue bar) and rough DP1004

(red open bar) after 1 h incubation at 37°C. No binding was observed with non-immunized serum (1:100) from naïve BALB/c mice. Data are represented as FI ± SEM of three independent experiments.
