# Supplementary material for: The Role of Host and Microbial Factors in the Pathogenesis of Pneumococcal Bacteraemia Arising from a Single Bacterial Cell Bottleneck
Source: PLoS Pathog. 2014 Mar 20;10(3):e1004026. doi: 10.1371/journal.ppat.1004026 (PMC3961388; doi:10.1371/journal.ppat.1004026)
Supplement: Figure S4 — Phagocytosis and surface marker characterization of different type of macrophages. Adhesion (A) and invasion (phagocytosis) (B) of four different pneumococcal strains (G54, TIGR4, D39 and DP1004) in primary spleen macrophages (SPM) isolates from C57BL/6 mice. Each symbol indicates a single value and results are represented as mean ± SD (n = 3). (C) Cytofluorimetric analysis of surface markers of spleen macrophages from C57BL/6 mice. A representative experiment was reported. Invasion of different pneumococci (G54, TIGR4, D39 and DP1004) in bone marrow macrophages (BMM) isolates from BALB/c mice (D) and in RAW264.7 macrophage cell line (F). Data (n = 6–12) are reported as scatter plots to better evidence the number of negative assay. Dashed lines indicate the detection limits for positive samples. Relative analysis of surface marker expression of bone marrow macrophages (E) and RAW264.7 (G) by flow cytometry. Data are represented as per cent of positive cells of representative experiments. (PDF) [file ppat.1004026.s004.pdf]

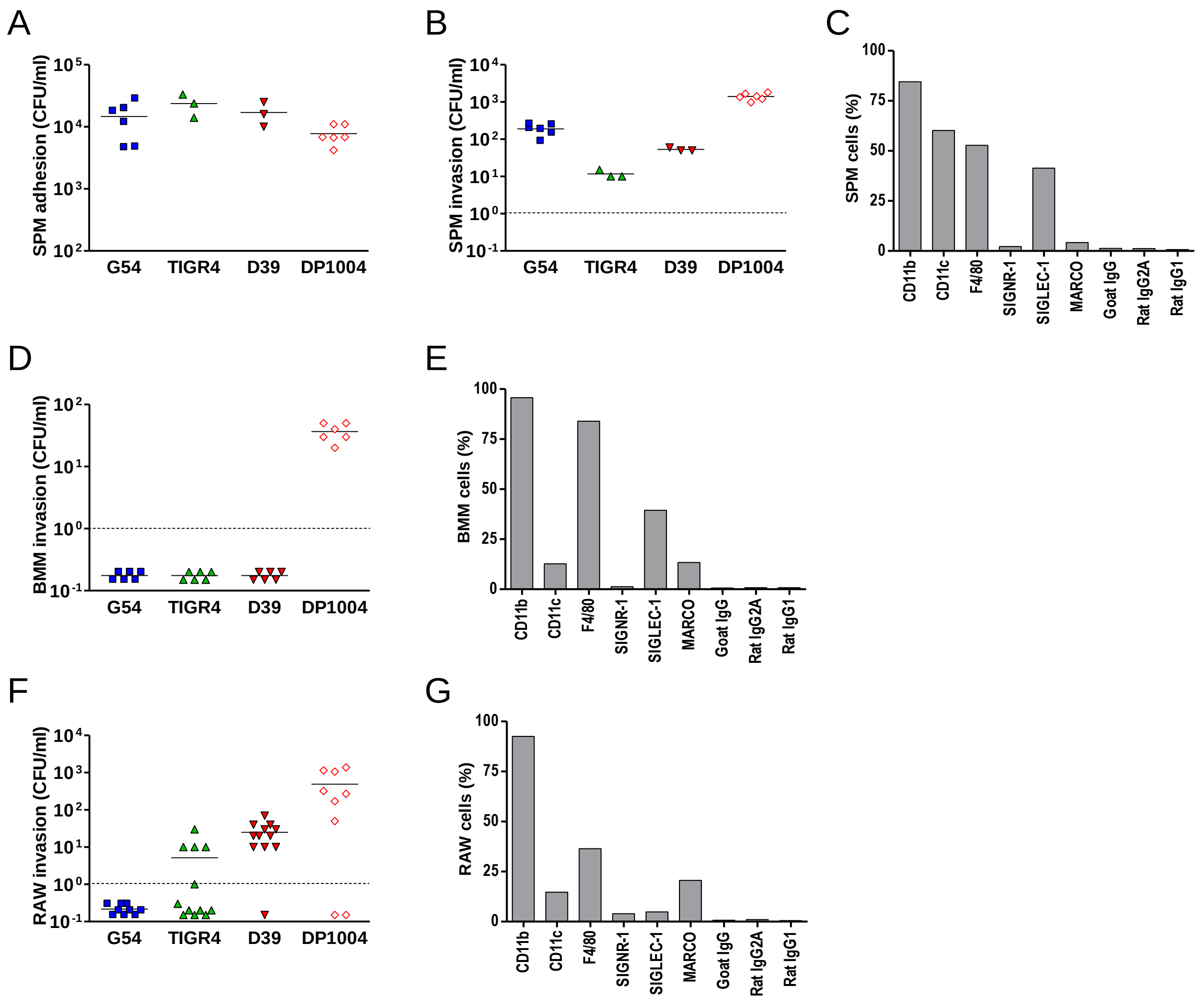

Figure S4. Phagocytosis and surface marker characterization of different type of macrophages. Adhesion (A) and invasion (phagocytosis) (B) of four different pneumococcal strains (G54, TIGR4, D39 and DP1004) in primary spleen macrophages (SPM) isolates from C57BL/6 mice. Each symbol indicates a single value and results are represented as mean ± SD (n=3). (C) Cytofluorimetric analysis of surface markers of spleen macrophages from C57BL/6 mice. A representative experiment was reported. Invasion of different pneumococci (G54, TIGR4, D39 and DP1004) in bone marrow macrophages (BMM) isolates from BALB/c mice (D) and in RAW264.7 macrophage cell line (F). Data (n=6-12) are reported as scatter plots to better evidence the number of negative assay. Dashed lines indicates the detection limits for positive samples. Relative analysis of surface marker expression of bone marrow macrophages (E) and RAW264.7 (G) by flow cytometry. Data are represented as percent of positive cells of representative experiments.
