# Supplementary material for: The Role of Host and Microbial Factors in the Pathogenesis of Pneumococcal Bacteraemia Arising from a Single Bacterial Cell Bottleneck
Source: PLoS Pathog. 2014 Mar 20;10(3):e1004026. doi: 10.1371/journal.ppat.1004026 (PMC3961388; doi:10.1371/journal.ppat.1004026)
Supplement: Figure S5 — Phenotype MicroArray of pneumococcal strains. Osmotic resistance of pneumococcal strains determined by Phenotype MicroArray. Bacterial metabolic activity determined in ethylene glycol (A), sodium nitrate (B) and sodium phosphate (C). Data were filtered using average height as a parameter (Biolog Omnilog-PM software). TIGR4 and the two TIGR4 derived challenge strains are in green, D39 in red and the mutants in black. (PDF) [file ppat.1004026.s005.pdf]

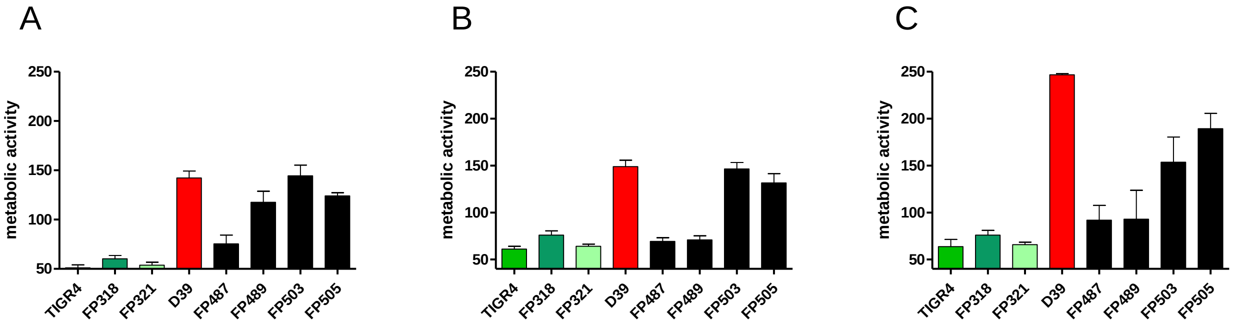

Figure S5. Phenotype MicroArray of pneumococcal strains. Osmotic resistance of pneumococcal strains determined by Phenotype MicroArray. Bacterial metabolic activity determined in ethylene glycol (A), sodium nitrate (B) and sodium phosphate (C). Data were filtered using average height as a parameter (Biolog Omnilog-PM software). TIGR4 and the two TIGR4 derived challenge strains are in green, D39 in red and the mutants in black.
