# Supplementary material for: The Role of Host and Microbial Factors in the Pathogenesis of Pneumococcal Bacteraemia Arising from a Single Bacterial Cell Bottleneck
Source: PLoS Pathog. 2014 Mar 20;10(3):e1004026. doi: 10.1371/journal.ppat.1004026 (PMC3961388; doi:10.1371/journal.ppat.1004026)
Supplement: Table S1 — Distribution of TIGR4 variants in monoclonal blood cultures. (PDF) [file ppat.1004026.s006.pdf]

Table S1. Distribution of TIGR4 variants in monoclonal blood cultures

	FP122<br>*ermB*	FP321<br>*aphIII*	FP318<br>*aad9**
24h	6	5	1
48h	4	6	2
72h	4	4	2

*challenge dose of FP318 was lower in one experiment
