# Supplementary material for: The Role of Host and Microbial Factors in the Pathogenesis of Pneumococcal Bacteraemia Arising from a Single Bacterial Cell Bottleneck
Source: PLoS Pathog. 2014 Mar 20;10(3):e1004026. doi: 10.1371/journal.ppat.1004026 (PMC3961388; doi:10.1371/journal.ppat.1004026)
Supplement: Text S1 — Model to establish the number of invading bacteria. Here we construct a statistical model to determine by which of two ways bacteraemia was generated. (PDF) [file ppat.1004026.s007.pdf]

SUPPLEMENRARY TEXT

Model to establish the number of invading bacteria. Here we construct a statistical model to determine by which of two ways bacteraemia was generated. We consider the following two alternative hypotheses of blood invasion: (i) establishment a bacterial population occurs starting from a single bacterium per event (note that this can also include the scenario of multiple invasion events; however, we assume that in each event only a single bacterium can invade); (ii) in each event a certain number of bacteria w (w>1) is required to overcome the “host defence” and successfully invade the blood (as in the previous case we allow for multiple invasion events as well). Testing the above hypotheses is equivalent to determining whether a single bacterium or several bacteria were involved in founding a bacterial population in the blood per invasion event. Our model was adopted from that described by Margolis and Levin [1], but differed inasmuch as the challenge was performed with three different isogenic variants, which required utilization of a permutation based approach, instead of a binominal distribution [1]. The model assumes that bacteria enter and establish a population in the blood at random by escaping from spleen tissue and the three variants are equally likely to invade.

Let us firstly consider establishing bacteraemia in a single mouse. We assume that the number of independent invasion events in a mouse, denoted by k, follows a Poisson distribution. This signifies that invasion events occur randomly with a constant average rate and independently of the time since the last event. Therefore the probability that k invasion events (k = 0, 1, 2, 3, …) occur in a mouse is given by:

$$P(k) = e^{-\lambda}\frac{\lambda^k}{k!},$$

where the parameter λ characterises the average rate of events. The particular case k=0 implies that no infection occurs. The value of λ can be calculated from the fact that $P(0) = e^{-\lambda}$. Using the data on the number of non-bacteraemic mice one can easily estimate the probability $P(0) \approx n_S / n$, where n is the total number of mice and n_s is the number of non-bacteraemic mice in the experiment. Thus we obtain

$$\lambda = -\ln[P(k=0)] \approx -\ln\left(\frac{n_S}{n}\right).$$

Let *w* be the number of bacteria that are responsible for founding a blood population in each invasion event (w = 1, 2, 3, …). Note that in each event the composition of isogenic variants within *w* can differ and it is assumed to be independent from that of the previous event. We denote *i* to be the number of variants observed in a single bacteraemic mouse. For convenience, we denote non-bacteraemic case by $i = 0$ so that i = 0, 1, 2 and 3 represents non bacteraemic, single, double and triple variant scenarios, respectively. Now we can calculate the probability that *i* variants will be observed in a mouse given *w* and *k*: $P(i|w,k)$. This is actually a conditional probability, i.e. it is the probability of observing an event in the case, where we are certain to have the given values of *w* and *k*. For example, $P(i=2|w=1,k=2)$ stands for the probability that a mouse has double variants in the blood if we are certain that a single cell is responsible for establishing the blood population in each of the events and the number of events is 2. It is easy to see that for any value of *w*,

$$P(i=0|w,k=0)=1 \text{ and } P(i|w,k=0)=0 \text{ for } i = 1, 2, 3,$$

in other words, without any invasion event (k=0), the probability to be infected is zero.

In the case of some invasion events ($k \geq 1$), the probability $P(i|w,k)$ can be calculated by using a standard permutation based approach, as in the case where we each time draw *w* balls from an urn containing balls of three colours and repeat *k* times with replacement and compute the probabilities of having single, double and triple colours. By doing this we assume that the number of bacteria in the stock (spleen tissue) is much higher than the number of escaped bacteria. In this case, the probabilities can be computed using the Bernoulli scheme with *wk* independent trials. We obtain $k \geq 1$

$$P(i=0\,|\,w,k)=0,$$
$$P(i=1\,|\,w,k)=\left(\frac{1}{3}\right)^{wk-1},$$
$$P(i=2\,|\,w,k)=\left(\frac{1}{3}\right)^{wk-1}(2^{wk}-2),$$
$$P(i=3\,|\,w,k)=1-P(i=1\,|\,w,k)-P(i=2\,|\,w,k)$$

Consider the case i=2, for instance. This corresponds to colonization by only two different bacterial variants after *k* events. Let us fix the two particular variants (e.g. choosing the first and the third variant). The given scenario will be possible in the case in any of *wk* ‘trials’ we should not have the second variant, with the probability to have such situation per trial being 2/3. This probability should be multiplied *wk* times by itself; we also need to subtract from it the probabilities of having only first or only third variants in all trials. This will give us $(2/3)^{kw} - (1/3)^{kw} - (1/3)^{kw}$. Finally, to

obtain $P(2|w,k)$ one needs to multiply the derived expression by the number of possibilities to choose 2 isogenic variants out of 3, i.e. by the factor of 3.

Next we need to consider all possible number of invasion events k, by summing up $P(i|w,k)$ over k (k= 0, 1, 2, 3, …) and taking into account the probabilities to have exactly k events (described by the Poisson distribution $P(k)$). Thus, for instance, the probability $P(2|w)$ means that exactly i variants are observed in a mouse if w bacteria are responsible for establishing the blood population in each invasion event. We can calculate $P(i|w)$ using a standard total probability formula:

$$P(i=0\,|\,w)=\sum_{k=0}^{\infty}P(k)\cdot P(i=0\,|\,w,k)=P(k=0),$$

$$P(i\,|\,w)=\sum_{k=0}^{\infty}P(k)\cdot P(i\,|\,w,k)=\sum_{k=1}^{\infty}P(k)\cdot P(i\,|\,w,k)\ \mathit{for}\ i=1,2,3$$

From our data we find that the probability that more than three independent invasion events occur in a mouse (k>3) is rather small and thus was ignored in the above calculation.

Given the total number of n mice, the expected numbers of individuals which are non-bacteraemic or infected with single, double or triple variants are given by

$$x_i=n\cdot P(i\,|\,w),$$

where w is the number of bacteria responsible for establishing the blood population in each invasion event. We assume that w is constant

Table 2 reports these expected numbers for w = 1, 2, 3, 4 and the observed ones at the 24 h time point when there were a total of 55 mice. Based on the estimation method of λ it follows that the expected number of non bacteraemic mice was equal to the observed one (n_s) this it is not presented in the table. The p-values of χ^2 goodness-of-fit test were calculated after collapsing the double and triple due to the small expected frequencies in these two categories when w=1 which may lead to unreliable result.

References

1. Margolis,E. and Levin,B.R. (2007). Within-host evolution for the invasiveness of commensal bacteria: an experimental study of bacteremias resulting from *Haemophilus influenzae* nasal carriage. J. Infect. Dis. *196*, 1068-1075.
